# Valorization of residual lignocellulosic biomass in South America: a review

**DOI:** 10.1007/s11356-024-33968-6

**Published:** 2024-07-02

**Authors:** Oscar H. Pardo Cuervo, Camila A. Rosas, Gustavo P. Romanelli

**Affiliations:** 1 Escuela de Ciencias Químicas, Facultad de Ciencias, Universidad Pedagógica y Tecnológica de Colombia UPTC, Avenida Central del Norte, Tunja, Boyacá, Colombia; 2 Centro de Investigación y Desarrollo en Ciencias Aplicadas “Dr. Jorge J. Ronco” (CINDECA-CCT La Plata-CONICET), Universidad Nacional de La Plata, Calle 47 No 257, B1900AJK La Plata, Argentina

**Keywords:** Typification, Biofuels, Energy, Platform molecules, Activated carbon, Pellets, Briquettes

## Abstract

Residual lignocellulosic biomass (RLB) is a valuable resource that can help address environmental issues by serving as an alternative to fossil fuels and as a raw material for producing various value-added molecules. To gain a comprehensive understanding of the use of lignocellulosic waste in South America, a review was conducted over the last 4 years. The review focused on energy generation, biofuel production, obtaining platform molecules (such as ethanol, hydroxymethylfurfural, furfural, and levulinic acid), and other materials of interest. The review found that Brazil, Colombia, and Ecuador had the most RLB sources, with sugarcane, oil palm, and rice crop residues being the most prominent. In South America, RLB is used to produce biogas, syngas, hydrogen, bio-oil, biodiesel, torrefied biomass, pellets, and biomass briquettes. The most studied and produced value-added molecule was ethanol, followed by furfural, hydroxymethylfurfural, and levulinic acid. Other applications of interest that have been developed with RLB include obtaining activated carbon and nanomaterials. Significant progress has been made in South America in utilizing RLB, and some countries have been more proactive in regulating its use. However, there is still much to learn about the potential of RLB in each country. This review provides an updated perspective on the typification and valorization of residual biomass in South America and discusses the level of research and technology being applied in the region. This information can be helpful for future research on RLB in South America.

## Introduction

Biomass is non-fossilized organic matter, which can originate from a spontaneous or induced biological process and contains stored chemical energy from the sun. It can be categorized into biomass residues and biomass remains. Biomass residues are primarily used for energy generation, while biomass remains can also be utilized in the production of other valuable products (Martí, 2018). These valuable products are often referred to as high-value added molecules (Das et al., 2021) or platform molecules (Quesada et al., 2014). Among different sources, lignocellulosic residues from crops are considered to hold the highest potential as raw materials for obtaining these molecules (Isikgor and Becer, 2015).

Residual lignocellulosic biomass (RLB) has played a significant role as a primary source of energy for humanity throughout history and continues to hold immense potential, with the ability to contribute approximately 14% of the world’s energy supply (Cáceres et al., 2016). Lignocellulosic biomass primarily originates from various sources, including forestry (wood residues), agriculture (plant materials generated in coffee, rice, banana farms, and other agricultural practices), industry (by-products resulting from industrial production processes), and urban areas (waste or garbage) (Afanasjeva et al. 2017). These diverse sources offer abundant opportunities for sustainable energy production and resource utilization.

RLB is a resource found all over the world, but in South America, it acquires special relevance due to the abundance of natural resources present in countries of this region and the residues generated by agricultural, forestry, and soil exploitation (Morandi et al., 2020; Jr et al., 2019). For example, in Chile, a forest biomass of 21.60 million dry tons per year (ts/year) is obtained, generating an average of 4 million tons per year (Mt/year) of firewood from plantations. Additionally, 7.97 Mt/year of agricultural residues are produced, with notable quantities including 2.50 Mt/year of beet crop residues, 1.80 Mt/year of wheat, 1.50 Mt/year of corn, and 1.10 Mt/year of potato residues. In Colombia, sugar mills annually produce 6 Mt of sugarcane bagasse from the 23 Mt of sugarcane, and nationally, 82.42 Mt/year of agricultural biomass residues are generated. The livestock and urban sectors produce 105.42 Mt/year and 0.17 Mt/year, respectively, of residual biomass. In 2015, Colombia generated 361,000 m^3^ of wood residues. In Ecuador, 1.47 Mt of sugarcane bagasse and 0.72 Mt of firewood were used for electricity generation, while approximately 18.23 Mt/year of agricultural residues are generated, and 0.22 Mt/year originates from the forestry sector. In Paraguay, 1610.83 ktoe of firewood, 700.30 ktoe of sugarcane products, and 584.14 ktoe of agroforestry residues such as cotton husks, sugarcane bagasse, and coconut shells were offered in 2016. In Peru, out of the 272 Mt of biomass, 256 Mt consist of firewood and 16 Mt are residues derived from agricultural, agroindustry, and forestry activities. Sugarcane bagasse (8.48 Mt) is available for bioethanol production. In Uruguay, approximately 0.20 Mt of rice husks, 0.26 Mt of wheat straw, and 0.04 Mt of sunflower husks are obtained annually. In Venezuela, nationally, 0.03 Mt/year of coffee residues, 2.85 Mt/year of sugarcane residues, 0.28 Mt/year of rice residues, 0.53 Mt/year of corn residues (straw and stalks), and 0.17 Mt/year of sorghum residues are generated (Vargas García et al., 2021).

South American countries play an important role in the global energy consumption landscape, particularly in the adoption and utilization of renewable energy sources, reflected in the growing implementation of solar photovoltaic and onshore wind technologies in several countries in the region. Brazil emerges as a pioneer in the adoption of renewable technologies, leading the implementation of solar photovoltaic and wind energy, surpassing its neighboring countries in installed capacity. Its major expansion in renewable energy, with notable amounts of 10 GW in solar photovoltaic energy and 4.1 GW in wind energy achieved by 2021, highlights its fundamental position in the region’s energy transition. Furthermore, initiatives such as the promotion of electric vehicles and the growing use of green hydrogen underline South America’s commitment to reducing carbon emissions and adopting sustainable energy solutions. With countries such as Brazil, Colombia, and Chile emerging as leaders in green hydrogen production, the region promises to foster innovation and drive global efforts towards a more sustainable energy future (Seminario-Córdova, 2023).

The participation of bioenergy in Paraguay’s total energy production is 40%, with 36% derived from hydroenergy and the remaining 36% from biomass. In Ecuador, in 2021, 93.2% of the electricity generated was renewable, primarily due to the continuous operation of hydroelectric plants. Wind and solar energy, along with geothermal and biomass, are other resources present in Ecuador, although their participation is still minor (around 1% of the mix). In Uruguay, 98% of electricity comes from renewable sources, mainly hydroelectric, solar, and wind. Biomass contributes 0.1% to the country’s electricity production. Venezuela hosts the fourth-largest hydroelectric plant in the world, which generates 72% of the country’s electricity. Brazil has maintained its position as the leader in installed renewable energy capacity in Latin America for several years, reaching 158 GW in 2021. Hydroelectric energy is the main contributor, representing 58% of all electricity generated in the country. Additionally, wind and solar photovoltaic energy play significant roles, with the former representing over 11% of the total capacity and the latter having an installed capacity of 23.1 gigawatts (GW). Biomass contributes approximately 9% to Brazil’s electricity matrix, with 15,320 MW installed (Mosquera, 2023). To our knowledge, the remaining South American countries have not reported the percentage of bioenergy participation in total energy production. However, the energy transition index (ETI) has been published, which is a metric that assesses a country’s progress towards a more sustainable and low-carbon energy system. A high ETI suggests that a country is moving towards a cleaner and more sustainable energy system, while a lower index indicates areas that need improvement. The reported ETI for South American countries is Brazil 65.9, Uruguay 63.6, Chile 62.5, Paraguay 61.9, Colombia 60.5, Perú 56.4, Bolivia 53.5, Ecuador 52.8, Argentina 52.0, and Venezuela 47.7 (World Economic Forum, 2023).

Currently, the main use of biomass is the production of biofuels and energy through mechanical, thermochemical, or biochemical conversion processes (Kalak, 2023). The largest volumes are concentrated in a few selected sectors, especially in the forestry industries in the form of sawmills and pulp and paper factories (International Energy Agency, 2022). The world’s ten largest biomass plants industrially exploit wood, sugarcane bagasse, agricultural by-products, wood waste, and peat (Roca José, 2016). Specifically, in South America, Novozymes, a Danish company, produces enzymes for the biofuels industry. Celulosa Arauco, a Chilean company, produces paper pulp and biofuels from wood. Braskem, a Brazilian company, produces chemicals and biofuels from sugarcane. Ecopetrol, a Colombian company, produces biofuels from sugarcane (Portafolio, 2014), and YPF, an Argentine company, produces biodiesel from vegetable oils (Huergo, 2018).

The production of clean energy from renewable sources of RLB has been promoted as it has multiple advantages for the environment (Sorita et al., 2020). Despite the great potential of RLB in South America, no document has been found that shown detailed information on its use in each country, whether for energy and biofuel generation, obtaining important platform molecules, or other types of materials. Most of the available information is focused on a specific country or topic, but there is no comparison that could serve as an indication of the level of technological and scientific development of South American countries in terms of the use of RLB to contribute to solutions for the imminent disappearance of petroleum sources and current environmental challenges.

Due to the aforementioned reasons, the main objective of this review is to present the use of RLB in South America and provide an updated and comprehensive perspective on the types of RLB used for the production of energy; biofuels; some platform molecules derived from it such as ethanol, hydroxymethylfurfural (HMF), levulinic acid (LA), and furfural (FF) (Bozell and Petersen, 2010); or other types of materials with specific applications. The information for each country was searched based on the PRISMA (Preferred Reporting Items for Systematic reviews and Meta-Analyses) methodology, which involves establishing search parameters, organizing, and selecting the information. The databases consulted were Google Scholar, ScienceDirect, Scopus, and Web of Science, and the reviewed period was 2019–2022. According to the reviewed information, Brazil was the country that carried out the greatest number of researches, followed by Colombia and Ecuador. A large amount of RLB used came from sugarcane residues (Vandenberghe et al., 2022), oil palm (Vanegas Escudero, 2019), rice (Cañon et al., 2022), and trees such as eucalyptus (Mendoza-Martinez et al., 2023). In South America, biogas (Ferreira et al., 2018; Tena et al., 2022; Maciel-Silva et al., 2019; Peres et al., 2019), syngas (Martinez et al., 2021; Furtado Júnior et al., 2020), hydrogen (Rojas et al., 2020), thermal energy (Martillo et al., 2020; Aseffe et al., 2019; Salgado et al., 2019; Pérez Arévalo and Velázquez Martí, 2020; Aguilar Romero, 2019; Machuca et al., 2020), bio-oil (Vanegas Escudero, 2019), torrefaction products (Talero et al., 2019; Vega et al., 2019), pellets (da Silva et al., 2020a; da Silva et al., 2022; de Souza et al., 2020), and briquettes (Mendoza Fandiño et al., 2020) were produced for biofuels and/or energy. Regarding the review on the production of platform molecules, it was found that Paraguay, Bolivia, Peru, Venezuela, and Uruguay had a lower participation in the production of ethanol, HMF, FF, and LA. Finally, it was found that RLB is used for the production of other products, including nanomaterials (Cidreira, 2019) and activated carbon, which are used for the adsorption of heavy metals and drug residues (Lima et al., 2019).

### General information on RLB

**Fig. 1:**
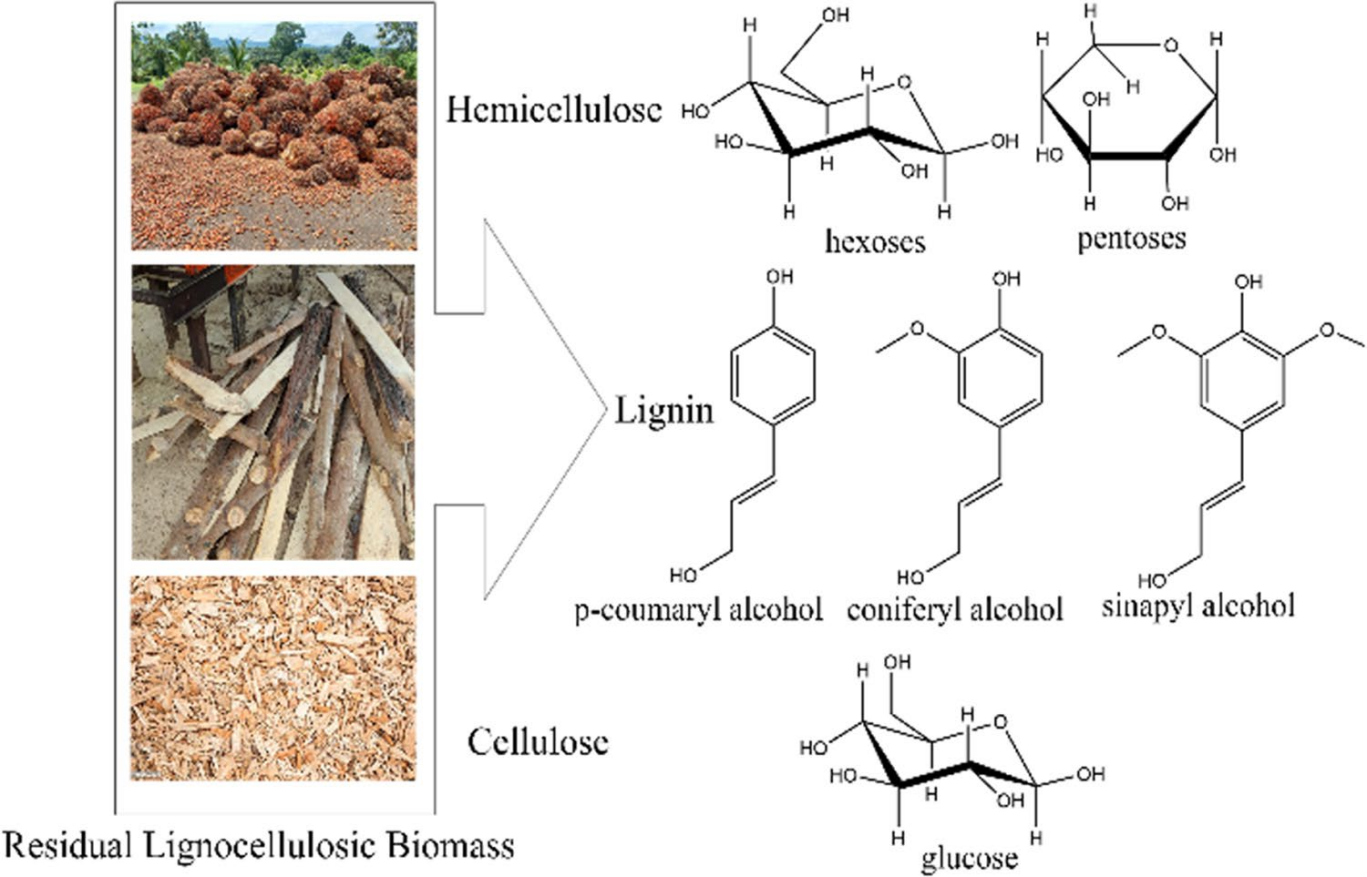
Components of lignocellulosic biomass

RLB is one of the most abundant resources in nature and is mainly composed of cellulose, hemicellulose, and lignin (Fig. 1). It constitutes an alternative to fossil fuels for obtaining energy and biofuels and as a starting point for obtaining chemical products of interest (US Energy Information Administration, 2021; Hernández-Beltrán et al., 2019; Jatoi et al., 2021).

### Types of residual lignocellulosic biomass

Biomass can be classified according to the type of residue it comes from. Accordingly, there is biomass from forest, agricultural, and industrial residues (Lago et al., 2018).

### Forest residues

This type of residue comes mainly from firewood and wood; it can be sawdust, tree felling debris, chips, and branches. They are obtained either in the extraction process or in the treatment of wood (Afanasjeva et al. 2017). This is one of the most widely distributed and therefore most abundant residues, and it is also considered renewable because as new crops are grown, new biomass is generated. Finally, it should be noted that although the main components of forest biomass are cellulose, hemicellulose, and lignin, their proportion varies according to the type of tree, the part from which the residues come, the type of wood (hard or soft) and environmental conditions (Elumalai and Pan, 2011).

### Agricultural residues

Agricultural residues refer to the leftover materials generated during agricultural practices, either during cultivation or harvesting, and vary according to the region in which they are produced, since some areas have a greater diversity of crops and this generates greater sources of biomass. This is the case of some countries, which have a large production of RLB, due to the amount of rice, banana, coffee, oil palm, sugar cane, corn, plantain, and sugar cane cultivations (Vargas García et al., 2021). Herbaceous and woody residues of high calorific value can be generated from these crops.

### Industrial residues

Industrial residues include by-products generated at the industrial level by the treatment of agricultural products; for example, rice husks or sugarcane bagasse. The composition and calorific capacity of each type of RLB is different, which means that pre-treatments are carried out according to the composition of the biomass and the products to be obtained.

### RLB conversion processes

To become the final product of interest, RLB requires physical, physicochemical, chemical, and biological pre-treatments that are applied depending on the type of waste and its composition (Das et al., 2021). Direct combustion is one of the most widely applied processes and has been used both in households and industry for cooking food, generating electricity in turbines and heating. The other existing processes are classified as thermochemical, biological, and chemical conversion (Osman et al., 2021).

### Thermochemical conversion processes

Thermochemical conversion is the controlled heating and/or oxidation of biomass as part of several pathways to produce intermediate energy carriers or heat. Thermochemical conversion technologies are classified by their associated oxidation environment, particle size, and heating rate. Prominent among these processes are pyrolysis, gasification, and torrefaction.

### Pyrolysis

This process is based on the degradation of RLB by subjecting it to high temperatures (300 and 600 °C), usually in the absence of air, in order to obtain fuels and other substances with very complex liquid phases in liquid, solid and gaseous states. This process can be carried out in fluidized bed, circulating fluidized bed, ablation, or cone jet reactors. A variation of pyrolysis is oxidative pyrolysis, in which oxygen is used to generate the combustion reactions necessary for biomass conversion (Rodríguez et al., 2019; Bertero et al., 2019; Galarza et al., 2021).

### Gasification

In this process, RLB undergoes high temperatures (800–900 °C) in the presence of a controlled amount of air to achieve incomplete combustion. As a result, gasification causes fewer environmental impacts compared to direct combustion (US Energy Information Administration, 2021; Duque Uribe, 2022). A gasifying agent is required to influence the composition and heat capacity of the produced synthesis gas, which is a mixture of combustible gases, CO, and H_2_ (Schmid et al., 2021). Gasification can be conducted using different types of gasifiers: fixed-bed gasifiers, which generate syngas with a high tar content (Galarza et al., 2021); cross-flow gasifiers, which yield syngas with a high carbon monoxide concentration; and fluidized-bed gasifiers, which produce syngas with a low tar content (US Energy Information Administration, 2021; González and Sandoval, 2020).

### Torrefaction

It is a type of mild pyrolysis carried out at RLB between 250 and 500 °C, under inert atmosphere. This process produces CO_2_, H_2_O, and torrefied material with high carbon content. Torrefaction reduces the water and volatile content of the biomass, improving its properties as a fuel, giving it greater energy density, hydrophobic behavior, and elimination of biological activity (Limousy et al., 2017).

### Biological conversion processes

#### Fermentation

Fermentation is a process in which RLB is converted into ethanol and gases such as methane and carbon dioxide through the breakdown of sugars by microorganisms in the absence of oxygen. To facilitate fermentation, a pretreatment of RLB is necessary in order to degrade lignin, which is inherently resistant to microbial degradation due to its high hydrophobicity and structural rigidity. A commonly used pretreatment method is enzymatic hydrolysis (Zoghlami and Paës, 2019).

#### Anaerobic digestion

Anaerobic digestion is a process that involves the decomposition of RLB to produce biogas, primarily composed of methane (used as fuel), and digestate, a two-phase residual material with various applications in agriculture. The process occurs in the presence of anaerobic bacteria, which carry out three stages: decomposition, conversion into organic acids, and conversion of the acids into methane. Different types of bacteria are involved in these stages, with acid-forming bacteria in the first two stages and both acetophilic and hydrogenophilic bacteria in the third stage (Afanasjeva et al. 2017).

### Chemical conversion processes

Chemical conversion, on the other hand, is not directly applied to RLB but is utilized on bio-oils produced from certain thermochemical treatments like pyrolysis. The bio-oil undergoes transesterification, either catalytically or non-catalytically, to obtain fatty acid methyl esters, which are then used to generate biodiesel. The reaction can utilize various alcohols such as methanol, ethanol, or butanol (Herguedas et al., 2012).

Table 1 shows the processes for RLB conversion, their strengths, and weaknesses, as well as their possible improvements.

**Table 1:** Strengths, weaknesses, and possible actions to improve RLB conversion processes.

TOP	CP	Strengths	Weaknesses	Possible improvements
Thermochemical	Pyrolysis	Widely applied for cooking, electricity generation, and heating in households and industries.	May release pollutants and greenhouse gases into the atmosphere, leading to environmental concerns.	Research and development of efficient catalysts to improve yields and quality of pyrolysis products. Exploration of new reactor configurations and operating conditions to maximize production of desired products and minimize formation of unwanted byproducts.
	Gasification	Produces synthesis gas (syngas) with fewer environmental impacts compared to direct combustion.	Requires high temperatures and a controlled amount of air, which may increase operational complexity and costs.	Improvement of gasification processes through development of more efficient and economically viable fluidized bed and fixed bed gasification technologies. Investigation of alternative gasification agents and biomass pretreatment methods to enhance quality and composition of synthesis gas.
	Torrefaction	Enhances biomass properties as fuel by reducing water and volatile content, increasing energy density, and improving hydrophobic behavior.	Requires energy input for heating, and the process may not be suitable for all biomass types.	Research into new feedstocks and pretreatment methods to optimize the torrefaction process and improve quality of the final product. Development of integrated torrefaction systems with energy production to increase overall efficiency and reduce operational costs.
Biological Conversion	Fermentation	Efficient conversion of RLB into ethanol and gases like methane and carbon dioxide. Utilizes microorganisms to breakdown sugars, making it a renewable and environmentally friendly process. Enzymatic hydrolysis pretreatment method can effectively degrade lignin, enhancing the efficiency of fermentation.	Requires pretreatment of RLB to degrade lignin, which adds complexity and cost to the process. Lignin is inherently resistant to microbial degradation, requiring specialized pretreatment methods.	Development and application of more efficient and specific enzymes for enzymatic hydrolysis, which could reduce costs and improve fermentation speed. Research into genetically modified microorganisms that can metabolize lignin more effectively, eliminating the need for costly pretreatments. Optimization of fermentation conditions such as pH, temperature, and substrate concentration to enhance process efficiency and productivity.
Biological conversion	Anaerobic digestion	Produces biogas primarily composed of methane, which can be used as fuel. Digestate, a residual material produced during anaerobic digestion, has various agricultural applications. Occurs in the absence of oxygen, reducing energy requirements and minimizing emissions of greenhouse gases.	Requires specific conditions and anaerobic bacteria for efficient decomposition, which may limit its applicability. Different stages of anaerobic digestion involve various types of bacteria, necessitating careful management and control. May produce odors and require proper management of digestate to prevent environmental impacts.	Enhancement of microbial diversity and activity through the use of co-cultures or microbial consortia specifically designed to maximize biomass degradation. Development of high-load anaerobic digestion systems capable of handling higher substrate concentrations and increasing biogas production. Investigation of new substrates and pretreatments to expand the range of biomass that can be anaerobically digested, including difficult-to-degrade lignocellulosic residues.
Chemical conversion		They allow the transformation of biomass into a variety of useful chemicals, such as alcohols, organic acids and hydrocarbons. Transesterification of bio-oils into fatty acid methyl esters (biodiesel) offers a renewable and environmentally friendly alternative to conventional diesel fuels. Various alcohols can be used in the transesterification process, providing flexibility in feedstock selection and process optimization.	Chemical conversion processes often require specific catalysts and operating conditions, which can increase costs and complexity. Transesterification reactions may produce unwanted byproducts or require additional purification steps, impacting overall process efficiency. The scalability and economic viability of chemical conversion processes may depend on factors such as feedstock availability, market demand, and regulatory requirements.	Development of more efficient and selective catalysts to enhance the speed and yield of transesterification. Research into new catalytic or non-catalytic methods that can reduce costs and improve product selectivity. Exploration of different reaction conditions such as temperature, pressure, and reactant molar ratio to optimize the process and minimize the formation of unwanted byproducts.
Direct combustion		Direct combustion is a simple and mature technology, offering potential for lower costs in both investment and operation compared to more intricate processes.It can leverage existing infrastructure from fossil fuel combustion, facilitating its implementation in certain cases.Can handle a variety of biomass types and forms without significant preprocessing.	Tends to have lower energy efficiency compared to other biomass conversion processes, resulting in lower energy yields.Tends to produce higher emissions of atmospheric pollutants compared to more advanced processes, which may require additional technologies for emission control and mitigation.	Implementation of advanced emission control systems to reduce atmospheric pollution.Integration of cogeneration systems to utilize residual heat and enhance energy efficiency of the process.Promotion of research in advanced combustion technologies to increase efficiency and reduce emissions.

*TOP* type of process, *CP* conversion process

The processes described above serve as the foundation for obtaining valuable products from RLB, known as platform molecules or high-value-added molecules. The US Department of Energy has compiled a list of these compounds that can be derived from biorefinery carbohydrates (Holladay et al., 2007). These molecules not only provide alternatives to petrochemicals and fossil fuels but also have significant potential. Table 2 presents the main processes used to obtain some platform molecule. It should be noted that all platform molecules presented in Table 2 are currently obtained on an industrial scale and are available on the market, although some of them are expensive. When prices were checked in US dollars at the Sigma Aldrich company, 5 g of 2,5-furandicarboxylic acid cost 285, 1 g of hydroxypropionic acid 71, 10 g of lactic acid 164, 1 L of glycerol 126, 10 mL of isoprene 342, 1 kg of levulinic acid 160, 1 kg of succinic acid 104, 1 L of ethanol 68, 500 mL of furfural 65, 25 g of hydroxymethylfurfural 538, 2.5 L of sorbitol 128, and 1 kg of xylitol 221. These prices are indicators of the need that exists to improve their production processes.

**Table 2:** Platform molecules and some production methods

Platform molecule	Method of obtaining	References
2,5-Furandicarboxylic acid	Oxidation of 5-HMF. Oxidation of furfural derived from lignin.	Hossain et al. (2017) Zhou et al. (2019)
Hydroxypropionic acid	Fermentation of glycerol and glucose fermentation	Takkellapati et al. (2018) Corma et al. (2007)
Lactic acid	Fermentation of sugars such as glucose, sucrose, and lactose.	
Glycerol	It is generated during the production of biodiesel.	
Isoprene	Its production from glucose is being worked on.	
Levulinic acid	Degradation of C6 sugars from lignocellulosic residues.	Dani (2016a)
Succinic acid	Fermentation of glucose from biomass.	Dani (2016b)
Ethanol	Biochemical transformation of biomass.	Bozell and Petersen (2010)
Furfural	Hydrolysis of lignocellulosic biomass to release pentoses.	Kabbour and Luque (2020)
Hydroxymethylfurfural	Dehydration of C6 sugars.	Bozell and Petersen (2010)
Sorbitol	Catalytic hydrogenation of glucose, at high temperatures and pressures.	Marques et al. (2016)
Xylitol	Hydrogenation of xylose derived from hemicellulose.	Ur-Rehman et al. (2015)

### Use of RLB for the production of biofuels or energy

**Fig. 2:**
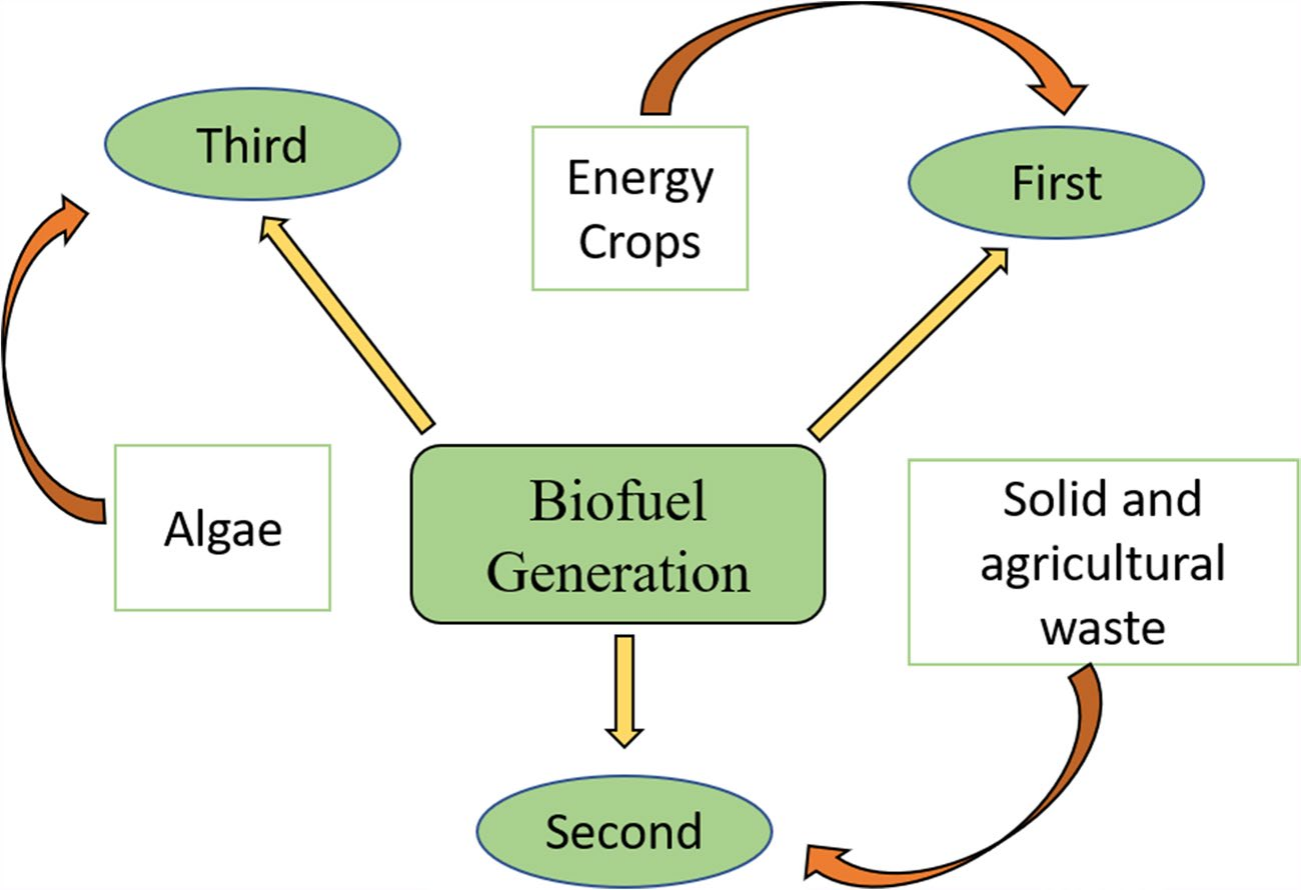
Classification of biofuels according to their source of obtaining

Due to the depletion of fossil fuel reserves and the associated greenhouse effect, it has become necessary to explore alternative sources for energy production. One promising option is RLB, which is considered a primary source of renewable energy (Kane et al., 2021). Various types of biofuels can be derived from lignocellulosic residues (Fig. 2). This review specifically focuses on second-generation biofuels derived from RLB, and the processes reported in Table 3 were carried out on a laboratory scale, through theoretical studies or feasibility studies.

**Table 3:** RLB conversion processes for biofuel and energy production reported in South America

Country	Process	Biomass source	Product	References
Brazil	Anaerobic digestion	Cassava stubble, acai peel and seed, sugarcane bagasse, corn, soybean, cassava and citrus stubble.	Biogas	Ferreira et al. (2018); Tena et al. (2022) Maciel-Silva et al. (2019); Peres et al. (2019)
	Gasification	Sugarcane bagasse, coffee husks and defective coffee beans, sugarcane straw, and oil palm stubble.	Biogas	Martinez et al. (2021); Furtado Júnior et al. (2020)
	Pyrolysis	Sugar cane straw, coffee stubble, rice husks, acai seeds, acerola, soursop waste, oil palm stubble, cassava stubble, and cocoa.	Syngas and hydrogen	de Souza Vandenberghe et al. (2022); Nunes et al. (2020) Santos et al. (2020); de Almeida-Couto et al. (2022) da Silva et al. (2020b)
	Briquetting	Coffee, rice, and sweet potato husks; citrus residues, pine sawdust and coffee husks.	Bio-oil and biochar	Martinez et al. (2019); Magnago et al. (2020) Faverzani Magnago (2022)
	Pelletizing	Elephant grass branches, eucalyptus sawdust and sugar cane bagasse, eucalyptus sawdust and coffee husk, leaves, and husks of corncob.	Briquettes	da Silva et al. (2020a); da Silva et al. (2022) de Souza et al. (2020)
	Direct combustion	Rice husks, forestry residues.	Pellets	Grotto et al. (2020); Lima et al. (2020)
	Transesterification	Defective coffee beans, oil palm bunches, and sugar cane bagasse.	Thermal energy	de Almeida et al. (2022); Batlle et al. (2021) Batlle et al. (2020)
Colombia	Anaerobic digestion	Cocoa mucilage, coffee mucilage, sugarcane bagasse, cassava, banana, corn, yam and rice stubble, mango husks, banana, sugarcane and oil palm stalks, banana peels, gulupa stubble, and rice straw.	Biogas	Vanegas Escudero (2019); Morantes Pacheco (2022) Parra-Ramírez et al. (2020) Posso and Mantilla (2019); Sagastume et al. (2022) Sagastume et al. (2021) Álvarez-Castillo and Ruiz-Carrión (2021); Amado et al. (2021) Durán Contreras et al. (2020); Mosquera et al. (2020) Durán-Aranguren et al. (2019a); Gutiérrez et al. (2020)
	Gasification	Coffee stalks and coffee husks, pine, teak and acacia branches, corn residues, sesame stalks, rice husks and cotton husks, pine sawdust, giant reed, cassava, and rice stubble.	Syngas, hydrogen, and electricity	Vega et al. (2019); Duque Uribe (2022) Durán-Aranguren et al. (2019b); Meramo et al. (2020) Garcia-Freites et al. (2020); Freites (2020) Gómez et al. (2021a); Gómez et al. (2021b) Quintero et al. (2021)
Colombia	Direct combustion	Banana stalks, rice and banana stubble, acacia, sugar cane straw, sugar cane bagasse, and banana peel.	Thermal energy	Sagastume et al. (2021); Gutiérrez et al. (2020) Serna-Jiménez et al. (2021); Vergara et al. (2020) Rivera-Cadavid et al. (2019)
	Pyrolysis	Oil palm residues, corn stubble, and sugar cane straw.	Bio-oil	Vanegas Escudero (2019)
	Torrefaction	Oil palm, pine, teak, teak and acacia bunches.	Torrefied material	Talero et al. (2019); Vega et al. (2019)
	Pelletizing	Sugar cane bagasse, coffee husks and rice husks, quinoa residues, and coffee husks.	Pellets	Manrique et al. (2019); Marrugo et al. (2019) Suárez-Rivero et al. (2019)
	Dark fermentation.	Cocoa mucilage.	Hydrogen	Rojas et al. (2020)
	Briquetting	Acacia sawdust.	Briquettes	Mendoza Fandiño et al. (2020)
Ecuador	Pyrolysis	Oil palm stalk, Jatropha curcas branches, corn stubble, oil palm bunches, and banana rachis.	Biogas, biodiesel, and bio-oil	Pérez Arévalo and Velázquez Martí (2020); Tombo et al. (2020) Medina et al. (2019); Meneses (2021) Streitwieser et al. (2021)
	Direct combustion	Corn stover, cocoa husk, dried corn, banana, African palm, banana and plantain leaves and pseudo stems, teak leaves, and palm kernel husk.	Thermal energy	Martillo et al. (2020); Aseffe et al. (2019) Salgado et al. (2019); Pérez Arévalo and Velázquez Martí (2020) Aguilar Romero (2019); Machuca et al. (2020)
	Gasification	Corn stubble and sugar cane bagasse.	Syngas	Aseffe et al. (2021); Poma (2019)
Ecuador	Anaerobic digestion	Peels, leaves and stems of banana, cocoa, banana, and banana mucilage.	Biogas	Solano-Apuntes et al. (2022) Zambrano-Gavilanes et al. (2021) Ponce et al. (2020)
	Pelletizing	Jatropha curcas leaves, rice husks, and coffee husks.	Pellets	Arteaga Quintana (2020); Ramírez et al. (2019) Orejuela-Escobar et al. (2021) Salgado et al. (2020)
	Compaction	Corn stubble	Briquettes	Delgado et al. (2020)
	Torrefaction	Oil palm mesocarp fiber.	Torrefied material (solid)	Salgado et al. (2020)
Argentina	Anaerobic digestion	Sugar cane bagasse	Biogas	Denaday et al. (2020)
	Pyrolysis	Pine sawdust and peanut shells	Bio-oil and biogas	Bertero et al. (2019); Galarza et al. (2021) Fermanelli et al. (2019)
	Gasification	Peanut shells, rice husks, and rice husks	Syngas	Leiva Butti et al. (2020)
Chile	Pelleting	Eucalyptus and pine sawdust, hazelnut shells, cherry pits, olive pits, corn cobs and grape. branches, wheat straw, oat straw, and grass straw.	Pellets	Rocha et al. (2020); Azocar et al. (2019) Murillo et al. (2021a)
	Roasting and pelletizing	Agroforestry residues.	Pellets	Vallejo et al. (2021)
	Hydrothermal carbonization and pelletizing.	Rape and pine residues, rapeseed bran, oat hulls and pine sawdust, pine sawdust, corn husks, and oat hulls.	Pellets	Silva et al. (2022); Monedero et al. (2019) Murillo et al. (2021b)
	Anaerobic digestion	Pomace and grape stalks.	Biogas	Montalvo et al. (2020)
	Gasification	Pine sawdust.	Syngas	Casas-Ledón et al. (2019)
	Hydrothermal carbonization.	Grape pomace and nut shells, pine sawdust, and rapeseed residues.	Hydrocarbon	Garrido et al. (2021); Vallejo et al. (2022)
	Direct combustion	Olive pomace, fruit pits, and vineyard prunings.	Thermal energy	Fernández-Puratich et al. (2021)
Uruguay	Direct combustion	Grape, olive, soybean, pine, eucalyptus, soybean stubble, wheat, rice, malting barley, corn, sorghum, grape pomace, stalks, and olive pruning.	Thermal energy	Pena et al. (2019); Dieste et al. (2019) González (2020)
Bolivia	Direct combustion	Sugarcane, soybean, corn, rice, sorghum, sunflower, and pine sawdust	Thermal energy	Morato et al. (2019)
Venezuela	Microwave heating	Sawdust waste agglomerated with corn starch	Briquettes	García-Escalona et al. (2019)
Paraguay	Direct combustion	Woody residues	Thermal energy	Ministerio de Obras Públicas y Comunicaciones de Paraguay, 2019

**Fig. 3:**
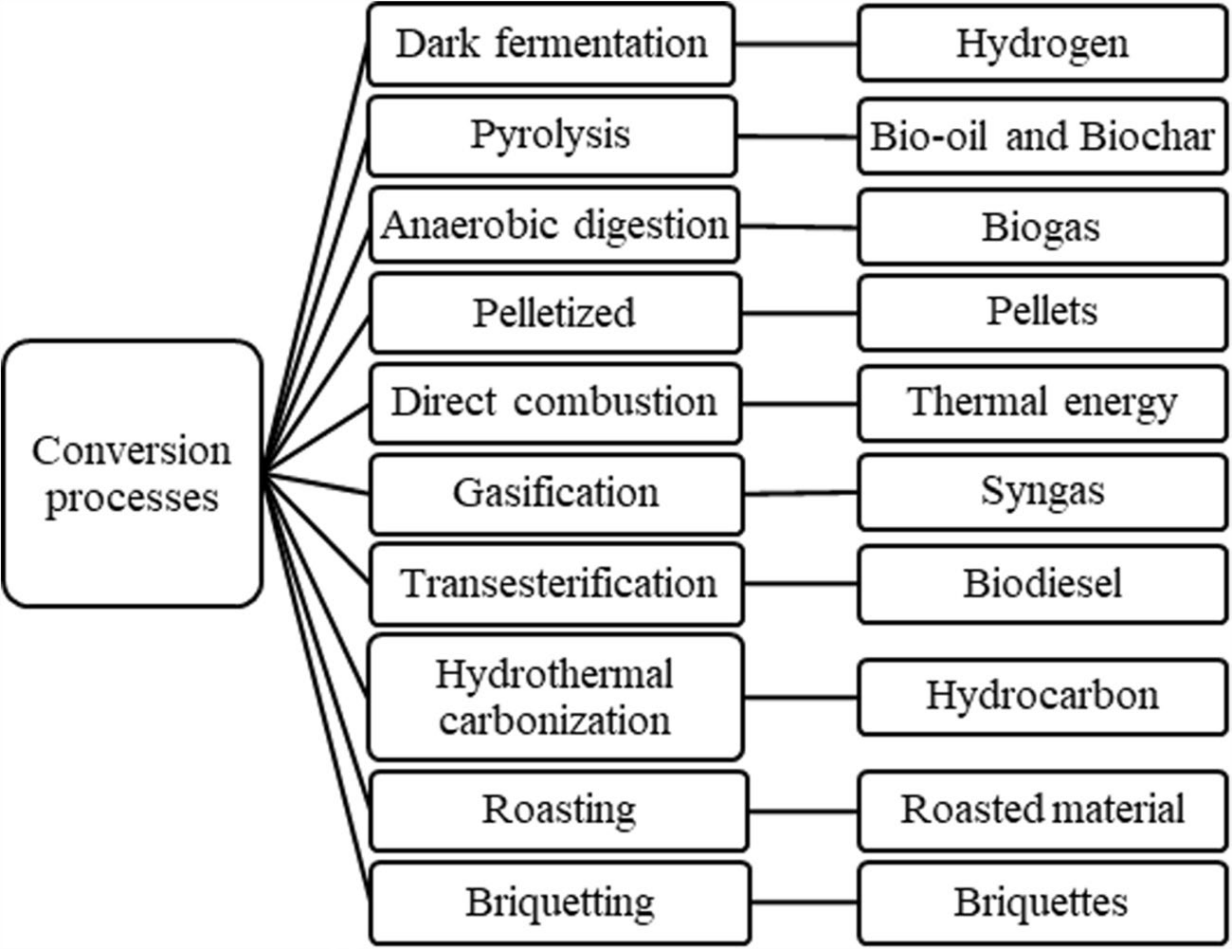
RLB conversion processes and main products

The RLB has certain disadvantages such as (a) loss of organic matter from the soil, (b) the energy invested may not be compensated, and (c) treatment difficulty due to the structure of lignin, characterized by its heterogeneous and rigid composition (Sosa et al., 2019). As a consequence of the last inconvenience, it is necessary that the biomass be treated prior to the conversion processes. Among the most implemented pre-treatments are chemical hydrolysis, enzymatic hydrolysis, hydrothermal treatment, and Organosolv (Yoo et al., 2020). After pre-treatment, the conversion process is applied to obtain the biofuel or energy. Figure 3 shows the conversion routes reported in this review.

The following section provides a description of how each South American country utilizes RLB to generate biofuels or energy. It is important to note that although ethanol derived from biomass is a biofuel, the results pertaining to it will be presented later (“Ethanol”) as it serves as a platform molecule for the production of other desired products.

Brazil’s energy matrix is characterized by its diversification, with sources including hydropower, wind energy, and biomass. The latter plays a crucial role in a country with extensive land area, biodiversity, and agricultural activities serving as its economic foundation. In terms of research focus on biofuel and energy production, thermochemical processes were the most commonly employed in Brazil (54%), followed by mechanical, biological, and chemical processes accounting for 21%, 14%, and 11%, respectively. Table 3 demonstrates that sugarcane residues are the most versatile among the investigated lignocellulosic materials, as they can be subjected to various processes, yielding a wide range of products. Additionally, it is worth mentioning that countries like Brazil and Colombia have conducted research on RLB in recent years with a particular focus on hydrogen.

Similar to Brazil, Colombia possesses a wide range of agricultural residues that can be converted into valuable products. Among these, hydrogen production is particularly notable, achieved through conventional gasification and dark fermentation processes. These complex processes involve the use of biomass as a substrate for obligate anaerobes and facultative anaerobes to act upon in the absence of light (Kamran and Fazal, 2021). Additionally, Colombia’s bioenergy conversion routes are distributed among thermochemical processes (51%), biochemical processes (36%), and mechanical processes (13%). Sugarcane, oil palm, and rice crop residues were the primary feedstocks utilized for bioenergy and/or fuel production (Table 3).

Ecuador, on the other hand, effectively utilizes its multiple sources of RLB through seven different conversion processes. Thermochemical processes such as pyrolysis, direct combustion, gasification, and torrefaction are particularly suitable for dry residues, offering higher energy yields without the need for pre-treatment. However, the energy yield decreases when these processes are applied to wet biomass, as it requires prior drying, resulting in increased energy consumption that may surpass the energy generated from the biomass, rendering the processes economically unfeasible. In contrast, biochemical processes like anaerobic digestion utilize wet residues since drying is not necessary, reducing biomass processing costs. Table 3 shows that 62% of the reported RLB in recent years in Ecuador was treated through thermochemical methods, 19% through biochemical processes, and the remaining 19% involved pressure-based processes, such as producing solid biofuels like pellets or briquettes. These solid biofuels increase the density and calorific value of RLB, allowing for greater utilization. This indicates Ecuador’s inclination towards thermochemical processes, likely due to their cost-effectiveness and lack of reliance on advanced technologies.

Argentina is progressively reducing its dependency on fossil fuels and has emerged as one of the top 10 biofuel-producing countries in 2021 (Statista, 2021). The country has also undertaken initiatives to generate clean energy from biomass, focusing on processes that minimize greenhouse gas emissions and combat climate change. Notable projects in Argentina include PROBIOMASA and RenovAR, both aiming to produce thermal and electrical energy from biomass (Coelho et al., 2020). Table 3 shows the processes employed to obtain biofuels and energy. It is worth mentioning that the reported RLB conversion routes in the past 4 years include anaerobic digestion, pyrolysis, and gasification. It is important to note that the biofuels derived from RLB were reported at a laboratory scale, with potential applications.

Chile has conducted extensive research utilizing RLB through various conversion processes. Similar to Ecuador, Chile has implemented different processes for residue treatment. However, Chile distinguishes itself by having the highest number of reports in the past 4 years specifically related to the production of solid biofuels, such as pellets. These pellets are of particular interest due to their high calorific value resulting from densification. A significant portion of the research efforts in Chile has focused on pellets production from single types of residues or mixtures of multiple residues. It is noteworthy that densification or pelletizing, in some cases, was combined with other processes such as hydrothermal carbonization or torrefaction, allowing the biomass to attain the required moisture conditions for solid biofuel production.

As shown in Table 3, numerous studies have emphasized the utilization of thermochemical processes. These processes predominantly involve biomass derived from forest crops. In contrast, a smaller proportion of research has focused on anaerobic digestion processes. Although less commonly employed, anaerobic digestion processes hold potential due to their technological simplicity and low energy consumption.

Uruguay, like other countries in South America, has implemented bioenergy production processes utilizing forest and agricultural biomass. However, it is important to note that the energy production in Uruguay primarily relies on energy crops rather than forest residues, which can potentially lead to deforestation. Nonetheless, in recent years, diverse types of RLB have been generated, including grape, olive, soybean, pine, eucalyptus, soybean stubble, wheat, rice, malting barley, corn, sorghum, grape pomace, stalks, and olive pruning. These residues have been subjected to thermochemical treatment, specifically direct combustion, to obtain thermal energy (Table 3).

In Peru, research has been conducted to assess the availability of agricultural resources and forest products, aiming to estimate the potential utilization of various residues for different applications such as energy generation and the production of composite materials (Sari et al., 2021).

Bolivia, despite being a small country with limited energy diversification, has the potential to enhance its energy resources through the utilization of RLB. While Bolivia possesses multiple biomass sources, only a fraction of them have been used for power generation in the past 4 years. The identified sources of residual lignocellulosic biomass include sugarcane, soybean, corn, rice, sorghum, sunflower, and pine sawdust. Direct combustion has been employed to generate thermal energy from these biomass sources (Table 3). It is important to note that while direct combustion is a common and widespread process, it can lead to environmental issues due to the emissions of sulfur, nitrogen oxides, hydrogen chlorides, dioxins, furans, and metals.

Venezuela, known for its heavy dependence on fossil fuels, particularly oil, has minimal available information regarding the use of RLB in the past 4 years. However, there is a report on the production of briquettes from sawdust waste agglomerated with corn starch (Table 3). Briquettes are a form of biofuel similar to pellets, but they have larger dimensions and are subjected to higher pressures during production. Forest residues and stubble are suitable materials for briquettes due to their dryness. Similar to pellet production, briquetting can be combined with processes such as pyrolysis, combustion, or torrefaction to treat different types of biomass. Pellets, small compressed cylinders of organic material such as sawdust or wood shavings, offer high energy density, low moisture content, and ease of storage and transportation, although they may entail a higher initial cost and reliance on supply. On the other hand, briquettes, compact blocks of similar material, are more versatile in terms of raw materials, durable, and less prone to damage during transportation, but they may have variable moisture content, affecting their combustion efficiency, and their handling and storage may be less convenient due to their size and weight. Ultimately, the choice between pellets and briquettes will depend on individual needs and the local availability of suitable raw materials and combustion equipment.

Paraguay heavily relies on hydroelectric power and biomass, but it should be noted that the biomass primarily comes from forestry crops, which poses sustainability challenges for the processes. Limited reports are available on the utilization of RLB in Paraguay. Woody residues have been used to obtain thermal energy, (Table 3) and the epicarp and mesocarp of *Acrocomia aculeata* have been utilized to extract oil and produce biodiesel through transesterification (Ovelar et al., 2019).

In South America, the utilization of RLB for biofuels and energy production shows a diverse range of technological advancements across different countries in the region. While Brazil diversifies its energy matrix with an emphasis on thermochemical processes and the versatility of sugarcane residues, Colombia stands out for its focus on hydrogen production and the wide range of agricultural raw materials employed. Ecuador, on the other hand, prioritizes thermochemical methods for waste treatment, minimizing processing costs by utilizing wet biomass in biochemical processes. Argentina is reducing its dependency on fossil fuels through initiatives focused on thermal and electrical energy production from biomass, while Chile distinguishes itself for its emphasis on solid biofuel production, especially high-calorific-value pellets. Uruguay, Bolivia, and Venezuela show potential to increase their energy resources through RLB, with predominant methods being direct combustion and briquette production. Lastly, Paraguay, though heavily reliant on hydroelectric power and biomass, still faces sustainability challenges in utilizing its forest resources. Together, these countries demonstrate a wide range of strategies and approaches in harnessing RLB to address their energy and environmental needs.

Overall, the utilization of RLB for biofuels and energy production shows varying degrees of advancement across South American countries, with each nation adopting different approaches based on its resource availability, technological expertise, and sustainability goals.

### Use of residual lignocellulosic biomass to obtain platform molecules (ethanol, HMF, FF, and LA)

Residual lignocellulosic biomass holds the potential for conversion into a diverse range of industrially valuable molecules. When these molecules are derived from biomass, they are referred to as bio-based chemical building blocks (De Jong et al., 2020). The US Department of Energy (DOE) has compiled a list of such molecules, and notable additions by Bozell and Petersen include ethanol, furfural (FF), and 5-hydroxymethylfurfural (HMF), which are widely reported in the literature, commercially produced, and considered to have significant potential as major building blocks (Bozell and Petersen, 2010).

The objective of this section is to present, organize, and discuss the research conducted in South American countries regarding the valorization of RLB into platform molecules, specifically ethanol, HMF, FF, and levulinic acid (LA), in recent years, in order to establish how each country has approached its production.

### Ethanol

Ethanol is a molecule of significant interest in this research due to its immense potential as a biofuel and as a platform molecule for various industrially important products, including ethylene, acetic acid, ethyl acetazte, and butanol (Rossi et al., 2021). It can be obtained through two main processes: synthesis gas from gasification or via the fermentation process. The fermentation process is the most commonly used method and typically involves three stages: (a) pre-treatment of the biomass, (b) saccharification (hydrolysis to obtain fermentable monosaccharides), and (c) fermentation, wherein microorganisms such as *Saccharomyces cerevisiae* or *Zymomonas mobilis* are employed to produce the desired product. Fermentation can be conducted simultaneously with saccharification or separately, with the former generally resulting in higher ethanol yields (Takkellapati et al., 2018).

Brazil is globally recognized as one of the leading ethanol-producing countries, which prompted several studies during the literature review period. In one of the initial studies, rice straw was utilized as the raw material, undergoing pretreatment with hydrolysis in subcritical water—a green process—under three different temperatures: 180 °C, 220 °C, and 260 °C. The optimum temperature of 220 °C yielded the highest amounts of fermentable sugars (1.54 g glucose, 3.41 g cellobiose, 7.81 g xylose, and 4.85 g arabinose per 100 g of biomass), which were subsequently used to produce ethanol (Abaide et al., 2019). Lachos-Perez et al. (2020) employed green processes to extract flavanones from orange peels through subcritical water treatment at 150 °C, followed by sugar extraction via hydrolysis at 200 °C, resulting in 13.44 g of glucose and 11.94 g of fructose per 100 g of biomass, providing potential fermentable products for bioethanol production. Sorita et al. (2020) reported the utilization of peanut shells, employing *Bacillus stearothermophilus* and *Saccharomyces cerevisiae*, to produce 16.11 g/L of ethanol. Additionally, sugarcane residues were subjected to a reactor with an inert nitrogen atmosphere to prevent oxidation reactions. A sequential process of enzymatic hydrolysis and fermentation with *Saccharomyces cerevisiae* was carried out, resulting in the production of 24.6 L of ethanol (de Oliveira et al., 2020). de Souza Vandenberghe et al. (2022) used cocoa hulls as a source of RLB, which are a common residue in the processing of this fruit. In their research, two processes are highlighted to generate ethanol; in the first one, an alkaline and hydrothermal pre-treatment was carried out and then fermentation with *Saccharomyces cerevisiae*. In the second, an alkaline pre-treatment was also carried out, but in this case, the fermentation was performed with the microorganism *Zymomonas mobilis*. Furtado Júnior et al. (2020) pretreated sugarcane bagasse with a steam explosion and dilute sulfuric acid, then performed a saccharification process and finally fermentation obtaining 149 L of ethanol/Ton of biomass. Finally, Uchôa et al. (2021) proposed the utilization of three banana residues (pulp, peel, and pseudostem) in a biorefinery, by means of a fermentation process with a co-culture of *Pachysolen tannophilus* and *Saccharomyces cerevisiae*, estimating an ethanol production of 0.4 g/g of biomass.

Colombia ranked second in terms of RLB sources reported in recent years, but only two studies focused on second generation ethanol production. Castro Pena et al. used three coffee residues (pulp, mucilage, and stems). Although the article does not specify the process for obtaining ethanol, it does describe the supply chain, including waste collection from each department, transportation to collection centers, and processing at the plants. The study estimated that the mucilage, pulp, and stems could produce 58.37 L/t, 25.17 L/t, and 240 L/t of ethanol, respectively (Castro-Peña et al., 2019).

In a different study, Meramo et al. (2020) carried out a computational study to obtain bioethanol through a waste reduction algorithm. This system considers atmospheric and toxicological aspects of the processes involved. The analyzed biochemical pathways were modeled and simulated using the Aspen Plus ® software package. The analysis was based on cassava residues and the mixture of cassava residues with plantain rachis. In the case of cassava, enzymatic hydrolysis and ABE (acetone-butanol-ethanol) fermentation were proposed, which is carried out with strict anaerobes and not facultative like conventional fermentations, and for the cassava-rachis mixture, acid hydrolysis and fermentation with *Zymomonas mobilis* were proposed. The simulation indicated that with the first scenario, 280 kg/h of ethanol could be obtained, together with 546.3 kg/h of acetone and 1305 kg/h of butanol, while with the cassava-rachis mixture, 2278 kg/h and 13865 kg/h of acetic acid could be obtained. The second scenario, in addition to generating a greater amount of alcohol with the mixture, generates a lower environmental impact, since there are fewer greenhouse gas emissions.

Understanding the supply chain is a crucial factor in RLB utilization. Success in residue treatment goes beyond the type of residue and the products that can be obtained; it also requires consideration of factors such as storage, processing, transportation, infrastructure, and costs, among others. Furthermore, this research highlights the importance of utilizing computational tools in RLB utilization processes as they can help optimize and improve these processes.

Ecuador is indeed one of the countries that provides a significant amount of information on the utilization of RLB. In one study, a mixture of corn, wheat, and barley stubble was used as the initial residues. The residues were first crushed and ground to reduce the particle size. Subsequently, they underwent enzymatic hydrolysis, saccharification, and fermentation processes, resulting in a 95% ethanol yield (Ronquillo Ponce, 2021). Ayala-Armijos et al. (2020) implemented rice dust as a source of residual biomass. The rice dust underwent enzymatic hydrolysis using enzymes produced by *Trichoderma* spp. The hydrolyzed product was then filtered and pasteurized to eliminate fungal remnants. Following these steps, fermentation was carried out for 72 h, resulting in an ethanol concentration of 5.1% v/v. A similar ethanol concentration of 7% v/v was obtained using banana peels, to which polyethylene glycol (PEG) was added to absorb the enzymatic hydrolysis inhibitors, thus optimizing the reaction (Bonilla et al., 2019). Additionally, *Jatropha curcas* residues have been successfully treated through simultaneous saccharification and fermentation processes to generate ethanol. The results demonstrated a maximum ethanol yield of 0.160–0.177 g ethanol/g dry biomass and cellulose conversion ranging from 57.7 to 63.9% (Sinche Arias et al., 2022).

The most commonly used residues in Ecuador come from cocoa cultivation, as the country is one of the main producers globally. Alvarez-Barreto et al. (2021) performed three types of pre-treatments on cocoa shells: acid (H_2_SO_4_), alkaline (NaOH), and autohydrolysis. The resulting liquors from the process were allowed to settle, then fermented, while the remaining solids underwent enzymatic hydrolysis with cellulase, followed by fermentation. This protocol yielded 28 g of ethanol per kilogram of treated biomass. In a purely theoretical study, the potential for bioethanol production from cocoa bark residues was estimated, for which a mathematical model was formulated in which it was proposed to perform acid hydrolysis on cocoa bark cellulose and its conversion to ethanol by fermentation to obtain degradation products such as glucose and then ferment with ethanogenic strains. The results showed that it is possible to obtain 8.28 × 10^6^ mL of ethanol/year in the country, considering the residues of each province (Sigüencia Avila et al., 2020). Starting from mucilage, which, being viscous and slightly liquid in texture, did not require pre-treatment and was directly fermented with 3 g of yeast/L, 25 g/L of ethanol were obtained (Delgado-Noboa et al., 2021). It should be noted that all the researches had in common that the fermentation was carried out with the same yeast: *Saccharomyces cerevisiae*, because it is a facultative anaerobic microorganism that has the ability to ferment glucose and does not present many demands in terms of nutrients (Bonilla et al., 2019). Similarly, enzymatic hydrolysis is the pre-treatment implemented in most of the researches, since it proved to be more effective than other types of hydrolysis.

In Argentina, sugarcane stalks underwent an acid-(H_2_SO_4_)-alkaline-(NaOH) pre-treatment and then an enzymatic hydrolysis with a Cellic CTec3 cocktail, reaching a 97% yield of glucose which is easily converted to ethanol (Kane et al., 2021). Mamaní et al. (2021) pretreated branches and pruning leaves of olive trees (*Olea Europea L.*) with lime, followed by enzymatic hydrolysis with cellulase/hemicellulase and detoxification with activated carbon to eliminate inhibitory compounds, such as polyphenols. Finally, they carried out fermentation with the yeasts: *Candida sake* BCs88, *Debaryomyces vanrijiae* BDv151, *Saccharomyces cerevisiae* BSc114, and *Saccharomyces cerevisiae*. The best ethanol yield was obtained with *Candida sake* yeast BCs88, obtaining 3.31 g/L ethanol per hour. Pine sawdust (*Pinus elliottii* and *Pinus taeda*) underwent a soda-ethanol pre-treatment to extract lignin and hemicellulose; then, two processes were carried out: the first consisted of hydrolysis with commercial enzymes Cellic CTec2 and subsequent fermentation with *Saccharomyces cerevisiae* IMR 1181. The second consisted of simultaneous saccharification and fermentation where *Saccharomyces cerevisiae* IMR 1181 was also used. The highest ethanol yield (92.50%) was obtained with the second process (Mendieta et al., 2021). A research was also conducted with grassland residues (*Spartina argentinensis Parodi and Panicum prionitis Ness*); however, only simulations were performed according to the amount of RLB. The results indicated that *Spartina argentinensis* was the species that would generate the highest ethanol yield (Sosa et al., 2019). Finally, Wagner et al. (2022) used beer spent grain as a source of RLB, which underwent acid pre-treatment (H_2_SO_4_), saccharification with *Trichoderma reesei* cellulase, and finally, fermentation with the ethanogenic strain *Escherichia coli* MS04. The bioethanol yield was 30 g/L per h and overall 251 L ethanol/t of spent grain. Considering the yields obtained, the RLB that Argentina should continue to explore further to increase ethanol production are those from pine and beer spent grain; especially the latter, since it is an innovative source that this country has used compared to all the others studied in this review, as no other country took advantage of this biomass.

Uruguay is characterized by the use of forest residues, due to the large number of hectares with trees to deforest. However, in some researches, it was found that the utilization of RLB is being chosen; for example, Rachid-Casnati et al. (2021) established the possibility of implementing a biorefinery, in which between 280 and 300 L of ethanol/t of Eucalyptus biomass (*Eucalyptus grandis*) would be obtained. Other authors, such as Guigou et al. (2019) performed an intensive pre-treatment of eucalyptus sawdust: some samples were initially treated with autohydrolysis at 170 °C and others with mechanical refining at 3000 rpm followed by Kraft pulping at 155 °C (3.4% alkaline loading). The remaining material, in both cases, was treated with enzymatic hydrolysis using Cellic CTec 2 (Cellulase). The researchers concluded that the best pre-treatment was pulping with soda as the enzymatic hydrolysis efficiency was more than 70% in the material subjected to this pre-treatment. Finally, after fermenting the residues with *Saccharomyces cerevisiae* they obtained 250 L of ethanol/t of sawdust.

One of the studies carried out in Peru was based on simulations of the amount of second-generation ethanol that could be obtained from residues of the most representative crops in the country. Considering the variables cellulose and hemicellulose mass percentage, type of crop, annual production, and percentage, the ethanol in kton/year that could be produced from sugarcane, rice, banana, yellow corn, oil palm, and asparagus would be (1.14 × 10^3^, 0.98 × 10^3^, 0.85 × 10^3^, 0.15 × 10^3^, 0.15 × 10^3^, and 0.25 × 10^3^), respectively (Retto-Hernandez et al., 2020). Naveda Rengifo et al. (2019) delignified rice hulls through steam explosions using a pressure of 1.0 MPa between 130–180 °C. This process succeeded in depolymerizing the lignin and reducing its content by 5.3%. Although the amount of ethanol was not determined, it was defined that the process of hydrolysis and subsequent fermentation of the lignocellulosic residues was facilitated, since the lignin did not prevent the degradation of sugars and microbial attack.

Bolivia produces ethanol from crops (first generation) such as sugarcane, but given the risk this represents for food security, it is reporting research on the use of agricultural and forestry residues to produce ethanol. Bagasse and orange peel were mechanically pre-treated with a shredder, then delignified with NaOH 0.1 M to obtain monosaccharides more easily. Then, some samples were subjected to acid hydrolysis (H_2_SO_4_ 5% v/v) and others were enzymatically hydrolyzed with amyloglucosidase from *Aspergillus niger* and α-amylase from *Aspergillus oryzae*. Finally, the hydrolysates were filtered and fermented with *Saccharomyces cerevisiae*. For orange peel, an ethanol yield of 0.029 cm^3^/g of orange peel and 0.031 cm^3^/g of orange peel was obtained with acid and enzymatic hydrolysis respectively; while for bagasse, a yield of 0.028 cm^3^/g of orange bagasse and 0.012 cm^3^/g of orange bagasse was obtained with acid and enzymatic hydrolysis respectively (Beltrán Siñani and Gil Bravo, 2021). Seeds of drupes of the pirul (*Schinus mole*) tree were ground and subjected to acid hydrolysis obtaining 14 g/L of monosaccharides and to enzymatic hydrolysis obtaining 3.2 g/L of monosaccharides. Finally, fermentation was carried out comparing native yeasts and common yeast *Saccharomyces cerevisiae*; with the former, an ethanol concentration between 200 and 250 mg/g was obtained, while with the latter only 125 mg/g (Solis et al., 2020).

It should be noted that in Bolivia, comparisons were made in the type of both pretreatment and fermentation to determine the best working conditions, and in addition to using *Saccharomyces cerevisiae* yeast, native strains were studied, demonstrating that they can be more efficient. Finally, acid hydrolysis was the best treatment for the different types of RLB, being the one that generated the best decomposition into fermentable monosaccharides.

In Chile, Paraguay, and Venezuela, most of the research on the use of RLB for ethanol production was conducted before the review period. In Chile, the objective of most research in recent years has been to obtain biofuels such as biogas or pellets. Although no recent information was collected on ethanol, in the years prior to 2019, there was use of residual biomass from pine and eucalyptus with which 262 L of ethanol/t of biomass were obtained (Vallejos et al., 2017; Marzialetti et al., 2014). Regarding Paraguay, it was reported that the country has 11 ethanol production plants; however, these are first generation plants, so the residues are not used, but rather the harvest of agricultural crops (Ministerio de Obras Públicas y Comunicaciones de Paraguay, 2019). Finally, in Venezuela, it was found that there is a study under development for the production of ethanol from lignocellulosic residues such as cassava bagasse and sugarcane straw (Melendez et al., 2021).

### 5-Hydroxymethylfurfural (HMF)

**Fig. 4:**
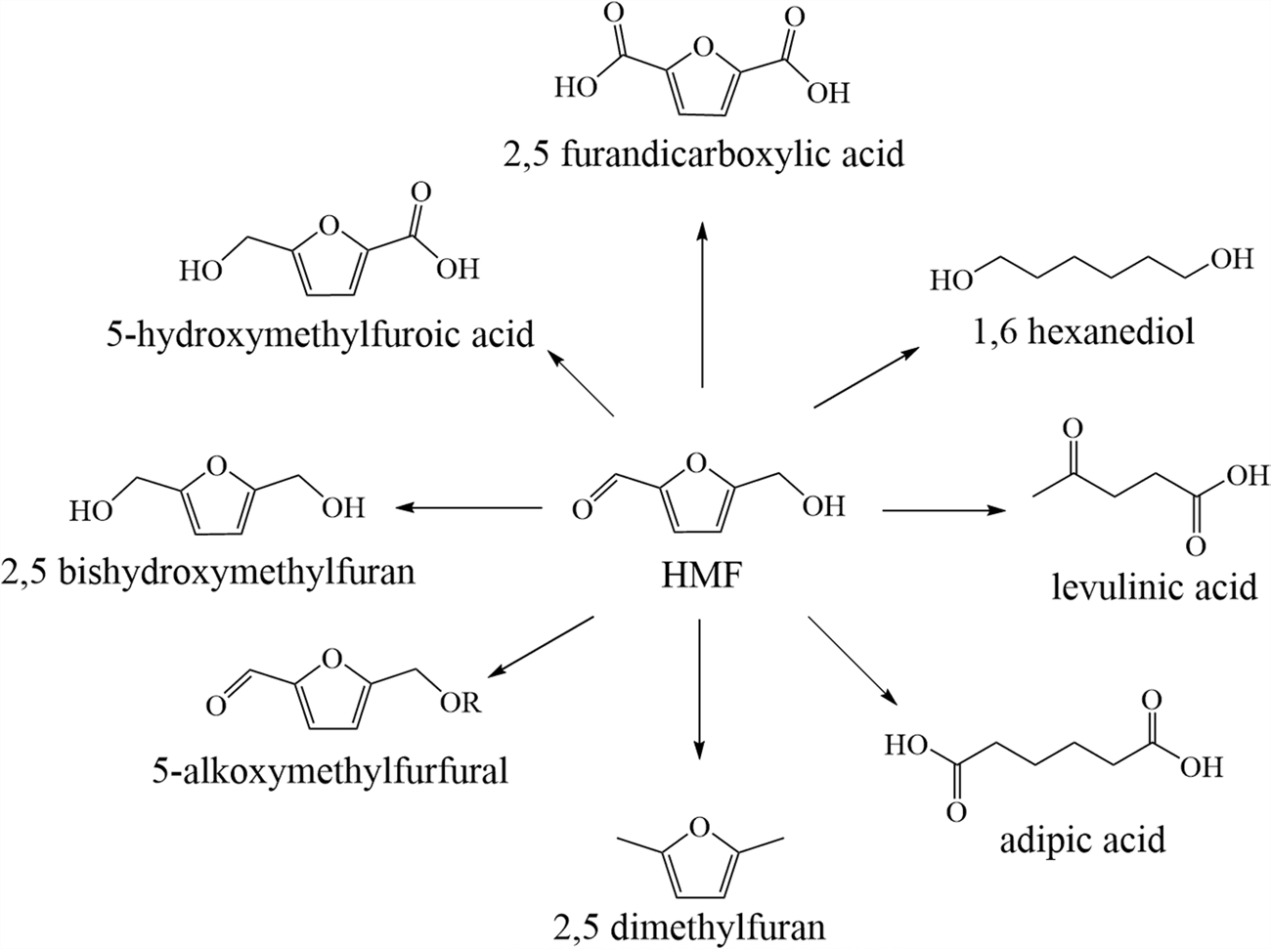
Some molecules of interest obtained from HMF. Adapted from Van Putten et al. (2013)

HMF is a very important platform molecule derived from furan because it is a precursor of multiple products of industrial interest such as polymers, liquid fuels, and pharmaceuticals (Fig. 4). There are two common routes to obtain HMF: the first involves hydrolysis of lignocellulosic biomass catalyzed by a Brönsted acid to obtain glucose, followed by isomerization of glucose to fructose by a Lewis acid and, finally, dehydration of fructose to HMF. In the second route, it is obtained by Maillard reactions that occur in foods (Van Putten et al., 2013).

In Brazil, sugarcane bagasse and peanut shells were treated with H_2_SO_4_ and 0.5 M HCl. From bagasse, they obtained 10 kg HMF/t biomass (0.99% w/w) yield, while peanut shells produced 572.6 g HMF/t biomass (0.057% w/w) yield. The low yield from peanut shells is due to its high lignin content and low monosaccharide content compared to bagasse (Godoy et al., 2020). On the other hand, Pereira et al. (2019) used calix[4] arene *p*-sulfonic acid as a catalyst in the dehydration of fructose present in biomass, obtaining a yield to HMF of 92% with 1 mol% of this acid. Other residues investigated in Brazil were commercial eucalyptus cellulose pulp and spent grain cellulose pulp from brewery, which was subjected to reaction using H_3_PW_12_O_40_/Nb_2_O_5_ (5% w/v) as catalyst obtaining a yield to HMF of 20% at 300 °C (Nogueira et al., 2020). Scapin et al. (2019) obtained 8.7% and 3.4% HMF from rice and soybean hulls, respectively, after pre-treatment with H_2_SO_4_ (72% v/v) and subsequent addition of the ionic liquid (1-n-butyl-3-methyl-imidazole bromide) to the hydrolysate.

As could be observed, the yields to HMF from RLB are low; however, it is possible to improve them by studying the reaction conditions and catalysts. Replacing conventional homogeneous catalysts such as H_2_SO_4_ or HCl by heterogeneous catalysts from a green chemistry point of view has a positive impact. This together with the implementation of new catalytic systems can contribute to more sustainable HMF processes.

HMF was produced in Colombia using the Organosolv process. Rose stems were ground, then treated with ethanol-water (1:1), glycerol-water (1:1), and ethanol-water-glycerol (1:2:1) at two different temperatures (130 °C and 200 °C) and times (30 and 90 min). The best yield at HMF was ~3%, obtained at 200 °C for 30 min, with the ethanol-water-glycerol mixture (Rincón, 2020). On the other hand, after hydrolysis of coffee stalk with 2% H_2_SO_4_, using toluene as solvent, NaCl as catalyst, atmospheric pressure and boiling temperature for 5 h, a yield to HMF of 5.6% was obtained (Pinilla Acosta, 2019).

In Ecuador, Ghysels et al. (2019) subjected walnut (*Phytelephas aequatorialis*) residues to the fast pyrolysis process at temperatures between 350 and 500 °C. The liquid obtained from pyrolysis was rich in levomannosan and 56% HMF.

In Argentina, peanut shells, rice husks, and wheat straw were valorized through pyrolysis under inert atmosphere at temperatures between 350 and 650 °C. In this process, the selectivity to HMF was 4.4%, 3.0%, 1.5% for peanuts, rice, and wheat, respectively (Fermanelli et al., 2020). On the other hand, the rice husk was subjected to an acid pre-treatment with 0.3% w/v H_2_SO_4_ at 150 °C and 5 atm, and then, the effect of temperature (127 °C, 160 °C, and 200 °C) was studied. In this way, HMF concentrations of 0.054 g/L, 0.062 g/L, and 0.410 g/L were obtained at each of the temperatures, respectively (Dagnino et al., 2019).

In Uruguay, pine (*Pinus taeda*) was treated with steam explosion at 200 °C for 10 min, obtaining 3.62 g/L of HMF (Yamakawa et al., 2022). On the other hand, Bonfiglio et al. (2019) used steam explosion with forest residues, in a semi-continuous equipment, i.e., releasing 15 bar pressure every 5 s. In the process, they heated to 200 °C for 15 min and obtained 0.54 g/L of HMF, while when heated to 170 °C for 10 min, they only produced 0.03 g/L of HMF.

In Chile, Peru, Bolivia, Venezuela, and Paraguay, no HMF production was reported in the databases consulted during the period reviewed.

### Furfural (FF)

**Fig. 5:**
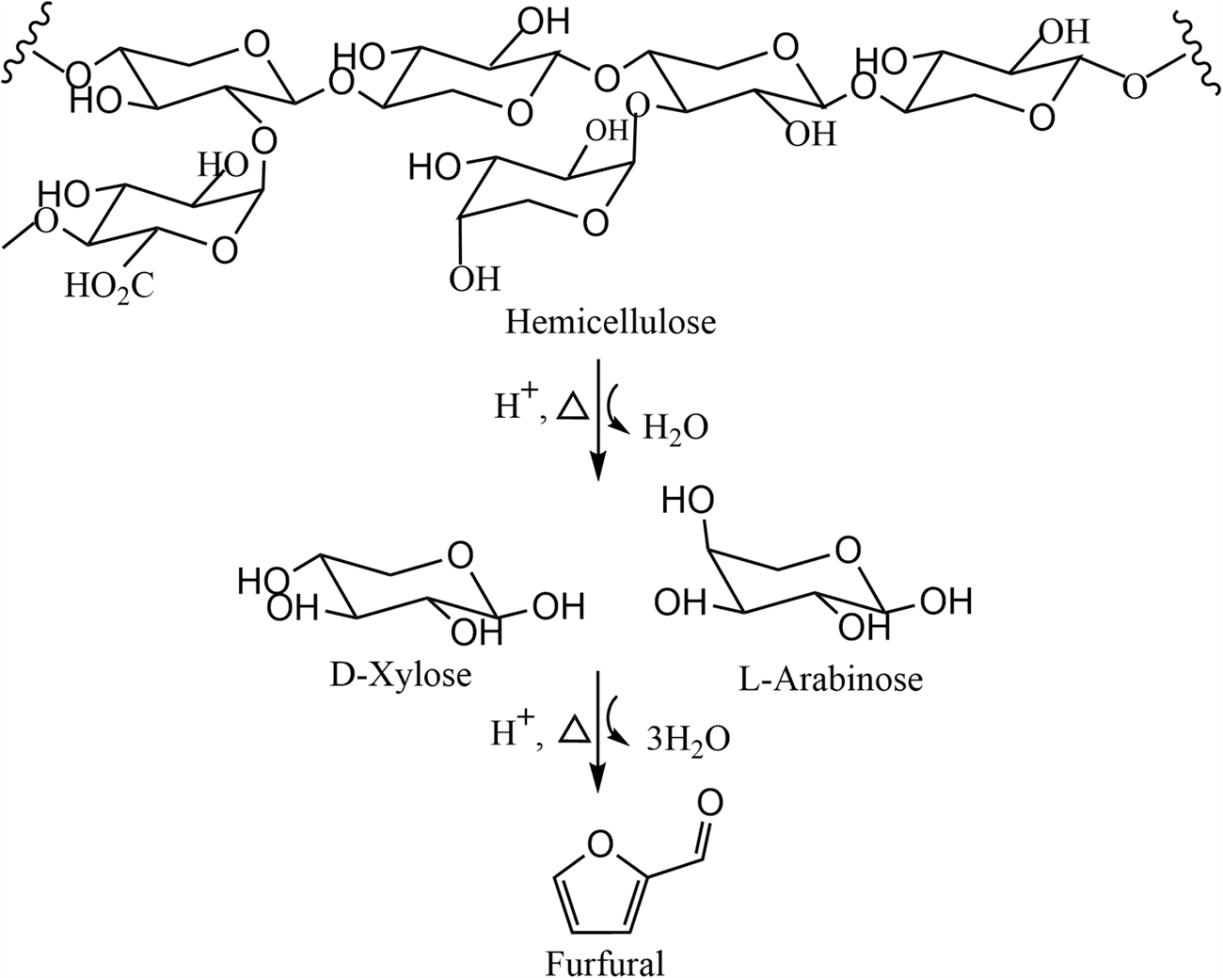
Route to obtain furfural from hemicellulose. Adapted from Kabbour and Luque (2020)

This compound is a derivative of the pentoses found in the hemicellulose of RLB (Fig. 5). The pentoses xylose and/or arabinose are dehydrated in acid medium to obtain FF. The commercial production of furfural has been known since 1922, where the Quaker oat company developed the first process from oat hulls, using liquid acid catalysts. The reaction involves the dehydration of the xylose molecule catalyzed by strong acids (H_2_SO_4_) and high temperatures (200–250 °C), presenting as a disadvantage the difficult separation of the catalyst and the formation of secondary products.

Multiple chemical species of industrial application such as levulinic acid, 2-furoic acid, furfuryl alcohol, furan, maleic anhydride, maleic acid, and succinic acid are obtained from this platform molecule (Kabbour and Luque, 2020).

In Brazil, rice hulls and soybean hulls were pretreated with H_2_SO_4_ (72% v/v); then, a reaction was carried out using the ionic liquid 1-n-butyl-3-methyl-imidazole bromide at 120 °C for 4 h, obtaining a yield of 34% and 59% from rice hulls and soybean hulls, respectively (Scapin et al., 2019). Bizzi et al. used acid hydrolysis (HNO_3,_ 4M) assisted by ultrasound (60 min sonication, 30 °C) obtaining FF yields of 3.6%, 4.9%, 6.1%, 6.2%, and 7.2% for rice husk, sugarcane straw, wood residues, yerba mate, and grass, respectively (Bizzi et al., 2019).

Among the information analyzed from Colombia, a review was found describing the processes for obtaining FF from RLB and the reactions to convert it into molecules of interest. For example, hydrogenation to generate furfuryl alcohol and oxidation to obtain maleic acid, furoic acid, and methyl furoate (Rodriguez et al., 2020). Laverde et al. (2019) reported an interesting route in which they used oil palm pulp, since it has a high content of cellulose and hemicellulose. An acid hydrolysis was carried out in a microwave extraction system using HCl (0.1M and 0.5 M) and CH_3_COOH (0.1M) at 180 °C and 160 °C and different reaction times. The best FF yield was 25% with 0.1 M HCl for 20 min at 180 °C. Pinilla Acosta (2019) performed hydrolysis with 2% H_2_SO_4_ on coffee grounds, consisting of 34.8% cellulose, 26.2% hemicellulose, and 22.9% lignin, obtaining a FF yield of 11% between 300 and 400 °C for 5 h. Finally, Rivera Marín reviewed the obtaining of FF from sugarcane bagasse, composed of 50% cellulose, 25% hemicellulose, and 25% lignin. The main advantage of this biomass is the high content of pentosans that hydrolyze to form pentoses that dehydrate to FF. The research determined that the suprayield process works best for treating residue. It should be noted that it is a continuous process that handles temperatures between 180 and 230 °C, and uses H_3_PO_4_ as a catalyst (Rivera Marín, 2021). The process basically consists of maintaining a reaction medium in a continuous state of boiling so that the furfural generated in the liquid phase is instantly transferred to the vapor phase. In the suprayield process, the boiling of the reaction medium is carried out in a different way, which consists of working at reduced pressure so that the liquid phase is continuously boiling at the reaction temperature.

Veliz Baquerizo (2019) ground and sieved sugarcane bagasse in Ecuador and then treated it with 15% H_2_SO_4_, obtaining 34.58 g of FF per kg of sugarcane. This research contributed to the valorization of bagasse in this country, since this residue is usually burned there. Another study also used bagasse, in this case together with rice husks and corn residues, which were subjected to hydrolysis with 8% H_2_SO_4_. The best yield at FF (2.75 g/L) was obtained from rice husk, followed by bagasse 2.59 g/L and finally corn 2.57 g/L (Murillo et al., 2020). Ghysels et al. (2019) applied the fast pyrolysis process between 350 and 500 °C to nut residues, reaching a maximum FF yield of 17%.

In Argentina, Celman et al. (2021) treated wood chips and carried out a FF recovery process by acid catalyzed dehydration (HCl 10% w/v) associated with a hydrodistillation obtaining a FF recovery of 5%. Fermanelli et al. (2020) carried out a pyrolysis process with different residues in a temperature range of 350-650 °C in a fixed-bed reactor fed by the heat of an electric furnace and with constant oxygen purge to avoid combustion. The selectivity to FF obtained was 7.5%, 6.5%, and 5.4% from peanuts, rice, and wheat, respectively.

Residual biomass from rice was used for FF production in Uruguay. Bariani et al. (2021) carried out a research relating three variables in a reactor: temperature (180–230 °C), H_2_SO_4_ (0.05–3% w/w), and time (1–105 min). The best yield (55%) according to the experimental design used was achieved at 200 °C, 0.10% w/w of acid, and 40 min of reaction, converting 6% w/w of rice husk into FF.

Pesantes Adrianzén and Tirado Ramírez (2020) in Peru performed acid hydrolysis with H_2_SO_4_ (6% w/v) at 122 °C for 100 min on rice crop residues, obtaining 2608 mg/Kg FF.

In Venezuela, Núnez et al. (2021) started from rice husks, which were ground, sieved, and macerated, and then subjected to reflux distillation for different times (8, 16, and 24 h). The FF yields obtained were 2.1%, 10.4%, and 9.4%, respectively. Boiling point (160.2 °C) and refractive index (1.52) at 20 °C were determined for the FF obtained.

### Levulinic acid (LA)

**Fig. 6:**
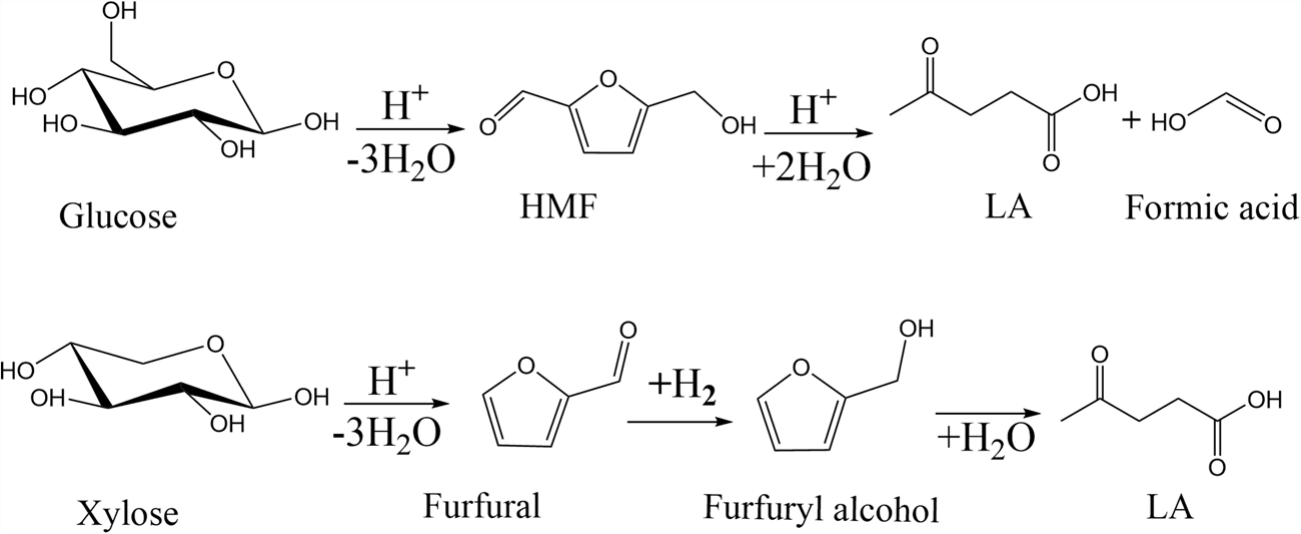
Route to obtain AL from glucose and xylose. Adapted from Santos et al. (2021)

LA is a keto acid that can be obtained biologically from cellulose. It was present in the first list of platform molecules made by the US Department of Energy because it is a promising molecule for generating fuels and chemicals such as textile dyes, polymer resins, and animal feed, among others. At a general level, cellulose and hemicellulose decompose into monosaccharides that in the presence of an acid dehydrate and reorganize into HMF (Fig. 6), which hydrates at the C2–C3 bond of the furan ring to obtain LA (Yan et al., 2015).

The main source of RLB used in Brazil for LA production was sugarcane bagasse. Albuquerque et al. (2022) implemented a novel technique to optimize enzymatic hydrolysis. They used a cocktail of enzymes from the fungi *Chrysoporthe cubensis* and *Penicillium pinophilum* at 121 °C with dilute acid (H_2_SO_4_ 5% v/v), although the yield of LA was not specified. The amounts of sugars obtained were determined: glucose 25.2 g/L and xylose 4.6 g/L which indicated a rapid degradation of glucan and xylan from RLB, which would result in higher amounts of LA. This research could be a reference for the use of other types of microorganisms in biomass treatment that can generate better LA yields and be more efficient than conventional ones. Silva et al. (2021) carried out a simulation of the LA production process to find a way to reduce the formation of humins (insoluble polymers), species that affect yield. The simulation was done in the Aspen Plus program, where two reactors were designed; in the first one, the rectification, reaction, and extraction sections were established, and in the second one, solids loading, cellulose conversion, catalyst loading, and inlet temperature. The authors found that the critical point for humin formation is the hydrolysis temperature, since high temperatures increase humin yield and decrease LA selectivity. Therefore, it was considered necessary to decrease the temperature and residence time in the hydrolysis and increase the catalyst loading, which, although it affected the yield to LA, decreased the amount of humins. Considering the above, an LA yield of 74 kg/ton of bagasse was obtained at 150 °C and with 5% v/v H_2_SO_4_ in 3 h. Finally, Lopes et al. (2020) also used computer tools to study the obtaining of LA, but in this case, they made predictions of the yield that could be obtained in the process using bagasse, rice husks and soybean straw. The authors implemented a biorefinery methodology in three stages. The first stage involved pre-treatment with 1.0% p/v H_2_SO_4_ at 121 °C for 90 min, using 15 g of each type of dry biomass. In the second stage, the solid fraction obtained in the hydrolysis was treated with NaOH at concentrations of 0.5% p/v for bagasse and 2.0% p/v for rice and soybean residues, under the same temperature and time conditions as stage one. This resulted in the production of insoluble solid residues and black liquor. Finally, the insoluble solid residues were subjected to a third stage, where a catalytic depolymerization of cellulose was carried out with 7.0% p/v H_2_SO_4_ at temperatures ranging between 150 and 190 °C for 75 min. The yields obtained experimentally were 60.5%, 65.2%, and 61.5% for bagasse, rice, and soybean, respectively. These values were close to those yielded by the prediction with the algorithm, which were 61.1%, 67.7%, and 61.4%, respectively.

It is important to note that in Brazil, they have advanced in the study of residual biomass, since they combine computational calculations with experimental work to improve the processes. This is useful to obtain better selectivity and yield in the products and allows saving reagents, catalysts, equipment, and reducing harmful substances generated, which favors sustainable chemistry applied to RLB.

In Colombia, Meramo Hurtado et al. (2021) performed simulations of LA production from empty banana bunches. They first proposed pre-treatment with H_2_SO_4_ 0.0011% w/w at 190 °C and 13 atm pressure, then enzymatic hydrolysis at 45 °C with a pressure of 1.66 atm and finally purification with a rate in the distillation towers of 5180 kg/h and 170 kg/h. The authors estimated a yield of 26% LA. Solarte-Toro et al. (2021) proposed a large-scale biorefinery model from avocado seeds and peels, the process consisted of saccharification to obtain liquid rich in hexoses, then using the acid resin Amberlyst 36 as a catalyst in a continuously stirred reactor at 160 °C at 5.5 MPa for 100 min and finally purifying the LA under distillation. With this simulation, a theoretical productivity of 23.83 kg of LA/h was reached. Although in neither of the two works were experimental processes carried out, thanks to the computational simulations it was possible to establish the best operating conditions when working with banana and avocado residues, which is useful for saving costs in reagents, equipment and time, together with the fact that the processes become green, reducing emissions and harmful by-products.

Clauser et al. (2020) in Argentina pretreated pine sawdust with 5% NaOH at 90 °C for 60 min to remove extractive solids from the biomass. Subsequently, for the extraction of hemicelluloses, the product was subjected to treatment with H_2_SO_4_ (7.5 g/L) at 150 °C for 90 min, where a liquor rich in hexoses was obtained. This liquor was concentrated by evaporation and taken to a reactor to dehydrate the hexoses to HMF in a process catalyzed by sulfuric acid with the same concentration as the previous step but for 30 min. Finally, the HMF was rehydrated to LA. Although the yield was not established in the research, a production of 156,000 t/year was estimated. An important factor in this report is that during the process, the liquor obtained during hydrolysis was reused in order to obtain a higher concentration of hexoses.

In Chile, researchers have studied the conversion of LA into other molecules of interest. During the reviewed period, an investigation was found that looked into the use of rhenium oxide catalysts for converting LA into gamma-valerolactone (Bassi et al., 2021).

In Uruguay, Benítez et al. (2022) carried out a chemical pre-treatment of pine sawdust with H_2_SO_4_ (2.2 g/L) in a reactor at 180 °C and 10 bar pressure. The solid fraction containing cellulose was then transferred to the reactor, where additional acid and steam were introduced at 190 °C and 12.92 bar. The liquid fraction containing LA was purified through an extraction process using FF, resulting in a 98% yield of LA.

In Ecuador, Peru, Bolivia, Venezuela, and Paraguay, no AL production was reported in the databases consulted during the period reviewed.

The use of RLB to obtain platform molecules such as ethanol, HMF, and FF has been the subject of research in several South American countries in recent years, with promising results. Brazil has stood out for its studies on ethanol production, achieving significant yields through pretreatment and fermentation processes of agricultural and forestry residues such as rice straw, peanut shells, and sugarcane residues. In Colombia, the exploration of second-generation ethanol production from coffee and rice residues has yielded encouraging results. Ecuador has succeeded in ethanol production from fruit residues such as banana peels and cocoa husks, employing enzymatic hydrolysis and fermentation. Argentina has achieved positive results using residues such as peanut shells and wood for ethanol and HMF production, through different pretreatment methods and catalysts. In Uruguay, the focus on forest residues such as eucalyptus has led to significant advances in ethanol production. In Venezuela, good results have been obtained in obtaining FF from rice residues through acid hydrolysis. Although research is limited in Chile and Paraguay, promising efforts have been reported for ethanol production from pine and eucalyptus residues in Chile, and agricultural crops in Paraguay. In summary, research in these countries shows a growing interest in valorizing RLB, with outstanding results that open new perspectives in platform molecule production. Brazil has made significant strides in utilizing sugarcane bagasse for LA production, optimizing enzymatic hydrolysis and conducting simulations to reduce humin formation, achieving a yield of 74 kg/ton of bagasse. In contrast, Colombia explored LA production from banana bunches and avocado residues through simulations, with estimated yields of 26% and a theoretical productivity of 23.83 kg/h, respectively. Argentina focused on pine sawdust, projecting a substantial production estimate of 156,000 t/year, while Uruguay achieved a 98% LA yield from pine sawdust through chemical pre-treatment and extraction processes. Chile investigated LA conversion into gamma-valerolactone using rhenium oxide catalysts. Notably, Ecuador, Peru, Bolivia, Venezuela, and Paraguay did not report LA production during the reviewed period, indicating varying levels of advancement in LA production across South American countries.

### Use of residual lignocellulosic biomass for other applications

The wide range of lignocellulosic residues available in South America make them a valuable resource for synthesizing new materials. Research has been conducted in several countries to develop novel materials or replace existing ones in fields such as pharmaceuticals, environmental science, electronics, and chemistry. Brazil, in particular, has spearheaded numerous research projects in the field of biomass-derived materials (Table 4). Cidreira (2019) performed a pre-treatment of delignification and bleaching to acai residues, where he centrifuged and dried the cellulose obtained to submit it to acid hydrolysis with a mixture (1:1) of H_2_SO_4_ (96%) and HCl (37%), obtaining cellulose nanocrystals (CNC) that were purified through ultrasound and lyophilization. The highest yield (11%) with a crystallinity of 45% was achieved with hydrolysis at 45 °C for 120 min. Ecological bio-adsorbents used for the removal of the yellow textile dye B2R were synthesized from chia seed residue. The process was based on extracting oil from the seeds and mixing it with textile wastewater containing the dye at a pH of 2 at 150 rpm. The best performance presented a bioadsorption of 70.95 mg/g and proved to be feasible for decontaminating water sources (da Silva and de Abreu Pietrobelli, 2019). On the other hand, Yabuki et al. (2019) using spent coffee grounds obtained a material to remove heavy metals from wastewater. They mixed the residues with agarose in a 10:3 ratio and heated at 80 °C to obtain a gel that was cut into discs to determine its performance as a binding phase for metals as Cd, Cu, Ni, Pb, and Zn. The results were satisfactory, due to the ionic strength of the material which was between 0.005 and 0.1 mol/L.

**Table 4:** Materials obtained from RLB in South America

Country	Residues	Treatment	Product
Brazil	Acaí	Hydrolysis	Cellulose nanocrystals.
		Pyrolysis and activation with KOH.	Activated carbon for adsorption of metal ions.
	Chia seeds	Extraction	Ecological bioadsorbents.
	Spent coffee	Agarose treatment.	Binding gel.
	Walnuts	Pyrolysis and activation with ZnCl_2._	Activated carbon to adsorb paracetamol.
	Macauba	Vacuum pyrolysis and activation with K_2_CO_3._	Activated carbon for adsorption of atrazine.
Colombia	Coffee cherry	Treatment with *Pleurotus ostreatus.*	Edible mushrooms
	Coffee pulp	Drying and crushing.	Mn bioadsorbents
	Avocado seed, blackberry stalks, and coffee residue.	Treatment with (NH_4_)_2_S_2_O_8._	Cr (VI) bioadsorbent
	Rice husks	Pyrolysis with controlled atmosphere.	Carbonaceous compound
Ecuador	Cane bagasse	Direct activation and hydrothermal carbonization.	Activated carbon
	Banana leaves	Fermentation with kombucha tea.	Bacterial nanocellulose
	Rice husks	Combustion	Blue-19 adsorbent
Argentina	Rice husk	Pyrolysis and extraction/evaporation.	Biocomposite films
	Pine sawdust	Hydrothermal treatment.	Mesoporous nanostructured material.
	Grape pomace and stalks	Microfibrillation.	Microfibrillated cellulose
	Yerba mate residue	Biochemical treatment with *Aspergillus niger.*	Bioactive compounds
		Solvent extraction.	Lipophilic antifungal extracts
	Rice husk	Extraction and treatment with AgNO_3._	Silver nanoparticles
Chile	Grape stalks and vine shoots.	Thermo-mechanical chemo-polishing.	Paper pulp
	Oat hulls, wheat straw, rapeseed stubble, and hazelnut hulls.	Autoignition of solvents.	Carbon nanotubes
	Wheat straw and corn husks.	Polishing.	Isolating material
	Corn and coffee	Carbonization and ZnO impregnation.	Activated carbon for As (V) and Pb (II) removal.
Perú	Red mombin seed, corn, coffee husk, mango and bean seeds.	Pyrolysis and impregnation with ZnCl_2._	Activated carbon for removal of norfloxacin and ofloxacin.
	Wheat straw and corn husks.	Crushing and drying.	Bioadsorbent of Cd (III) and Pb (II).
Uruguay	Rice husks	Carbonization and KOH	Activated carbons
Bolivia	Amazonian nut residues	Carbonization and KOH with nitrogen flow	With nitrogen flow

An important material, given its adsorption capacity, is activated carbon. In Brazil, they considered its properties and carried out multiple researches aimed at taking advantage of the RLB to obtain it. Lima et al. (2019) used walnut shells to obtain activated carbon which was used as an adsorbent to retain acetaminophen from water and hospital effluents. For the formation of activated carbon, they used ZnCl_2_ as an activating agent at 600 °C. A material with high surface area (1640 m^2^ g^−1^) and porosity was obtained, generating an adsorption of 411 mg/g, with which it can be inferred that the RLB used is a suitable raw material for the elaboration of activated carbons. On the other hand, Vieira et al. (2021) with the objective of mitigating contamination by atrazine, a common herbicide, developed an activated carbon from macauba residues by vacuum pyrolysis at temperatures between 400 and 600 °C, at a heating rate of 10–50 °C/min and with K_2_CO_3_ as activating agent. The synthesized carbon had a surface area of 1002.5 m^2^ g^−1^ and pores of 2.67 nm; in addition, it presented a high affinity for atrazine since it eliminated it by 98%. Queiroz et al. (2020) proposed to obtain activated carbon from acai seeds to remove metal ions in water. The seeds were washed, dried, and ground to particles smaller than 0.250 mm and sieved, then heated at 600 °C with N_2_ flow and impregnated with 50% KOH, heated again at 130 °C, and then at 850 °C and finally washed with 1 M HCl and dried. The material obtained had a surface area between 1462 and 2774 m^2^ g^−1^ and removed 86% of Pb, 69% of Fe, and 8% of Mg.

Research conducted in Brazil showed special interest in obtaining activated carbons for the removal of substances harmful to water sources, since 50% of the reports were based on the synthesis of carbons to eliminate different substances, including drugs and herbicides. Research carried out in Brazil showed special interest in obtaining activated carbons for the removal of harmful substances from water sources, since 50% of the reports were based on the synthesis of carbons to eliminate different substances, including medicines and herbicides, present in it. It should be noted that they used unconventional RLB sources for this purpose.

Table 4 shows the materials obtained from RLB in Colombia. Given the importance of coffee cultivation in the agriculture of this country, the residues generated in its production and harvest have been studied to mitigate the impact of leaving them in the open air to decompose without any treatment. González et al. (2020) determined the content of lignin (29%) and cellulose (48.4%) in the coffee cherry. Subsequently, they used it as a substrate for the cultivation of the Pleurotus ostreatus fungus. The cherry substrate was inoculated and incubated at a temperature of 24 °C, with 85% humidity and total darkness. After the process, it was observed that the biomass was adequate, since the primordia appeared after 15 days and the fungus was harvested after 20 days. These results demonstrate that the coffee cherry is a suitable substrate for the growth and development of Pleurotus ostreatus. Gómez Aguilar et al. (2020) used coffee fruit pulp to elaborate a bioadsorbent for Mn (II) from synthetic wastewater. First, the residue was characterized, establishing a composition of 30% cellulose and 19.3% lignin. Subsequently, it was dried directly in the sun for 15 days and crushed to obtain the adsorbent material with a particle size of 180 μm which presented a maximum adsorption capacity of 8.01 mg/g at pH 4.0, 100 rpm, 20 °C, and a contact time of 90 min.

Rangel et al. (2021) used avocado seed, blackberry stems, and spent coffee. Initially, they were crushed, washed, and dried; then, each of the samples was treated with (NH_4_)_2_S_2_O_8_ 0.1 M at 300 rpm and then heated at 30 °C for 12 h. They were later washed with water and dried at 80 °C to obtain activated solids. All the residue types showed adsorption capacity for Cr (VI), a toxic species that is mainly generated by industrial activities. The adsorption capacity was 1.4 mg/g, 9.7 mg/g, and 10.6 mg/g for avocado, coffee, and blackberry residues, respectively. Finally, and given that rice is a product that is generated in dozens of municipalities in the country, it was necessary to work with its residues, so its husk was studied to establish the capacity to generate carbonaceous compounds. Castro et al. developed a pyrolysis system with controlled nitrogen atmosphere where they subjected the biomass to a temperature of ~1000 °C in a range of 0 to 300 min, where they obtained a material with tubular morphology that could have applications in science and technology (Castro-Ladino et al., 2020). It is important to emphasize the importance of investigating each type of biomass in particular, as each has unique aspects and, therefore, different pre-treatments, treatments, and applications.

Table 4 shows the materials obtained from RLB in Ecuador. In this country, RLB was also used to obtain activated carbon. Valladares Ochoa (2020) conducted two procedures to produce activated carbon from sugarcane bagasse. In the first procedure, the bagasse was directly activated with NaOH solutions of different concentrations (1 M, 2 M, and 4 M) and then heated to temperatures between 500 and 600 °C. In the second procedure, the bagasse underwent hydrothermal carbonization pretreatment in a reactor at 200 °C for 12 h. The resulting solution was vacuum-filtered, washed, and dried, and then activated with NaOH solutions of the same concentrations used in the first procedure. The second procedure yielded a higher activated carbon yield, with 49.1% compared to 23% in the first procedure. Peñafiel et al. (2021) used sugarcane bagasse as a precursor for a ciprofloxacin adsorbent material. The bagasse was washed with water, dried at 60 °C for 24 h and ground: the product was tested in a fixed-bed column with the adsorbate and a removal of 78% was achieved with 3 g/L of biomass at pH 6–8. Meanwhile, Fiallos-Cárdenas et al. (2021) used banana leaf residue to create bacterial nanocellulose. The process began with obtaining bagasse extracts that were subsequently centrifuged and fermented with 10% kombucha tea, since it is suitable for the growth of *Saccharomyces* spp., *Zygosaccharomyces* spp., and *Brettanomyces bruxellensis*, followed by purification with NaOH 3M, and washing with distilled water. The yield obtained was 0.031 g bacterial nanocellulose/g fermentation medium, and the production rate was 0.11 g/L*h. Rice husk was used for the removal of blue dye 19. In this research, Zambrano-Intriago et al. (2022) carried out tests with untreated rice husk and its ashes at pH 2, an adsorbent dose of 1 g/100 mL and a time of 120 min. The studies showed that there was greater affinity between the dye and the surface of the untreated rice husk.

Table 4 shows the materials obtained from RLB in Argentina. Taverna et al. developed films based on tar microparticles loaded with alginate and eugenol. The tar was obtained from rice husks by pyrolysis carried out in a downdraft reactor at 485 °C for 5 h with an air flow of 16 L/min, obtaining 1.8% yield. Microparticles were synthesized with this tar using the extraction/evaporation method with dichloromethane and finally eugenol was added. The films were used as active packaging materials to improve the shelf life of canned foods (Taverna et al., 2022). Another use given to rice residues was to obtain mesoporous nanostructured materials. Carraro et al. (2021) prepared a mesoporous material for use as a catalyst in degrading endocrine disruptors by first washing and drying rice husks at 100 °C. The husks were then treated with 1M HNO_3_ and filtered, followed by another round of drying for 24 h and calcination at 800 °C. To extract silica, the calcined material was treated with 1M NaOH at 80 °C for 18 h and centrifuged, with the resulting supernatant used along with cetyltrimethylammonium to produce the mesoporous material.

On the other hand, González et al. (2022) used pine sawdust to obtain microfibrillated cellulose to make films and coatings. The process began with bleaching of the pulp, which was subsequently hydrated for 24 h and dispersed in a mixer, then refined in a laboratory mill and microfibrillated in a high-pressure homogenizer. The cellulose obtained showed high relative transmittance and short microfibrils.

Since one of the most produced residual biomasses in Argentina comes from vineyards, Meini et al. (2020) synthesized bioactive compounds from pomace of different types of grapes. The method consisted of subjecting the RLB to the fungus *Aspergillus niger* with tannic acid as a substrate to obtain tannase and gallic acid. The pomace was subjected to submerged fermentation and fungal growth occurred at 30 °C for 5 days. The pomace of the Pinot Noir grape variety produced the highest amount of gallic acid (1.5 mg/mL); no tannase production was reported. Palazzolo et al. (2022) performed lipophilic extracts of Cabernet Sauvignon, Malbec, Chardonnay, and Pedro Giménez grape stems in different solvents (hexane, diethyl ether, ethanol, and ethyl acetate) in order to determine their antifungal activity against *Aspergillus candidus*, *Penicillium chrysogenum*, and *Rhizopus* sp. The ethanol extract of Pedro Giménez grape showed the highest activity, which is associated with the presence of a higher amount of gamma-sitosterol, an antifungal compound.

Finally, Arreche et al. (2020) obtained silver nanoparticles from yerba mate residue. The procedure was based on heating aqueous extracts of the biomass at 70 °C for 30 min, centrifuging and mixing in a 1:10 ratio with a 1 mM solution of AgNO_3_ under magnetic stirring at 1000 rpm at 25 °C for 24 h. In the research, spherical, triangular, and hexagonal nanoparticles with a size close to 50 nm were obtained and showed antimicrobial activity against *Escherichia coli* and *Staphylococcus aureus.*

Table 4 shows the materials obtained from RLB in Chile. This country is known for its vineyards, which produce countless residues such as stalks and shoots, which were used in the research of Elissetche et al. (2020) in which they carried out a mechanical chemo-thermal pulping process on these residues in two steps. In the first, they treated them with Na_2_SO_3_ at temperatures between 130 and 160 °C for 60 min. In the second, they carried out mechanical refining and thus obtained pulp from the residue, which proved to be an environmentally friendly alternative to paper because it had a low chemical load. Other abundant residues in this country are oat hulls, wheat straw, rapeseed stubble, and hazelnut shells, which were studied to obtain carbon nanotubes through the solvent autoignition method in a muffle furnace. First were subjected to pyrolysis at 600 °C, and then, the autoignition was performed with hexane. The biochars obtained had nanotubes with diameters of 225 nm, 220 nm, 190 nm, and 290 nm from oat, wheat, rapeseed, and hazelnut residues, respectively (Hidalgo et al., 2020).

In this country, the utilization of RLB has also been directed to the area of construction materials. Rojas et al. (2019) developed an insulating material based on wheat straw and corn husk fibers using the biomass pulping method. The product showed good results in tensile and flexural tests compared with synthetic insulating materials synthesized from petrochemical substances.

In Uruguay, research was carried out on the use of RLB for applications in the technological and pharmaceutical fields. da Silva et al. (2021) prepared activated carbons from rice husks that were treated with a solution of HNO_3_ for 24 h to eliminate impurities, then rinsed and dried for 24 h at 105 °C. Carbonization was carried out at 400 °C over a period between 1 and 4 h, and KOH (40% wt/wt) was used as the activating agent. The carbon had a high specific surface area of 2412 m^2^ g^−1^, high electrical capacitance, and good energy and power density, which demonstrated that it is possible to obtain materials for calomel-saturated electrode applications from RLB.

Nicole et al. (2022) used rice husks to obtain nanocellulose (NC) for enzyme immobilization and imaging. The process started with semi-solid fermentation using *Trichoderma reseii* and *Phanaerochaete chrysosporium* which are microorganisms known to produce cellulases. Next the NC was obtained from the husk by a biological method using a microscale technique with simultaneous saccharification and fermentation (SSF) implementing *Trichoderma reseii* and *chrysosporium preinoculum* pellets for the process. The NC presented a yield of 55%, high stability, and lamellar structure of 10 nm. Finally, the nanocellulose-derived material was treated with hydrazinonicotinic acid, producing a compound useful as a radiopharmaceutical agent.

In Peru, research has been carried out to obtain materials that remove toxic substances from water sources. Table 4 shows the materials obtained from RLB in the country. Cruz et al. (2020) dried at 80 °C ground corn and coffee residues to obtain a particle size of less than 0.5 mm, then carbonized at 600°C for 2 h and impregnated with ZnO through the precipitation method. The obtained products proved to be able to remove As (V) and Pb (II) from water. Bednárek et al. (2022) prepared activated carbons from red mombin seed, corn, coffee husk, mango, and bean seeds in order to remove norfloxacin and ofloxacin from water. The residues were dried at 80 °C and ground; then, each was mixed with ZnCl_2_, pyrolyzed at 600 °C, cooled, and washed with 0.15 M HCl solution. Activated carbons obtained from red mombin seeds adsorbed metals from water more efficiently, followed by corn, coffee, mango seeds, and bean residues. Finally, Salazar-Pinto et al. (2021) sought to remove Cd (III) and Pb (II) from water, elements that accumulate and biomagnify. The methodology was based on bean residues (stems, leaves, and pods) that were crushed, sieved, and washed with water until a constant pH was achieved. They were then dried at 70–75 °C for 24 h. The tests showed a removal of 87.7% and 95.6% for Pb and Cd, respectively, evidencing a high capacity to remove these metals from water.

All the research in Peru was based on the production of materials capable of adsorbing different chemical species harmful to water sources. Note that they were based on different processes and used mostly zinc derivatives that act as Lewis acids in the treatment.

In Bolivia, a study was carried out in which biomass residues were used to mitigate the problem generated by CO_2_ as greenhouse gases. Serafin et al. (2021) started with Amazonian nut residues which were washed, dried, and crushed to obtain particles below 100 μm. These were then impregnated with the activating agent KOH for 1 h and dried for 19 h to be carbonized in a furnace with nitrogen flow, where the temperature flow was from 273 to 1073 K/min. The result was activated carbon with a surface area of 1624 m^2^ g^−1^, high microporosity (0.43 cm^3^ g^−1^), and with the capacity to adsorb 5.13 mmol CO_2_/g, which made walnut residues a potential tool to develop materials for environmental applications.

The utilization of RLB for various applications has been a focal point of technological advancement across South American countries. Brazil has led significant research efforts in this area, particularly in synthesizing new materials from diverse RLB sources. For instance, cellulose nanocrystals were derived from acai residues through pre-treatment and acid hydrolysis, while chia seed residue was utilized to develop ecological bioadsorbents for wastewater treatment. Additionally, spent coffee grounds were repurposed for removing heavy metals from wastewater by creating a gel material. Brazil also explored the production of activated carbon from walnut shells and macauba residues, demonstrating high adsorption capacities for substances like acetaminophen and atrazine. Similarly, Colombia investigated the potential of coffee cherry and pulp residues for applications such as edible mushroom cultivation and manganese bioadsorbents, respectively. Moreover, avocado seed, blackberry stems, and coffee residues were used to synthesize bioadsorbents for chromium removal, showcasing the versatility of RLB across different applications. In Argentina, rice husks were employed to develop biocomposite films and mesoporous nanostructured materials, while grape pomace and stalks were utilized for extracting bioactive compounds and lipophilic antifungal extracts. Chile focused on repurposing vineyard residues for paper pulp production and generating carbon nanotubes from various agricultural by-products. Furthermore, oat hulls, wheat straw, and corn husks were studied for producing isolating materials. Peru concentrated on producing activated carbons from corn, coffee, and bean residues to remove toxic substances from water sources, highlighting the importance of addressing water contamination issues. Additionally, Bolivia explored the potential of Amazonian nut residues for carbon sequestration to mitigate CO_2_ emissions. Overall, these endeavors underscore the diverse applications of RLB across South American countries, ranging from environmental remediation to material synthesis, thereby contributing to sustainable development in the region.

### Green economy and politics

Countries in South America are distinguished by their vast biodiversity, with some located in the Amazon basin, others in desert or semidesert regions, and some possessing rich wetlands and marine ecosystems (IRENA, 2024). This diversity strengthens the potential to decouple regional economic growth from fossil fuel consumption and promote a green economy based on cleaner energy sources (Marquez, 2012). Brazil has maintained its leadership in renewable energy installed capacity in Latin America for years, reaching 158 GW in 2021. However, other countries in the region are also actively working to harness their own natural resources. According to the 2022 report from the Latin American Energy Organization, Paraguay stands out for achieving completely renewable electricity generation, mainly thanks to its abundant water resources and plants like Itaipú and Yaciretá. Additionally, they are considering implementing green hydrogen projects for the transportation sector. Ecuador, although heavily reliant on hydroelectricity for its electricity generation, is looking to increase its share of other renewable energies, such as wind and solar, and is currently implementing a legal framework to promote investment in clean energies. Uruguay has successfully transformed its energy matrix, standing out for a high percentage of renewable energy, especially through hydroelectric, solar, and wind power. Its public-private partnership model has been recognized by international organizations. Colombia, despite its abundance of water resources for hydroelectric generation, is also exploring the potential of wind, solar, geothermal, and biomass energy to diversify its energy matrix. Finally, Venezuela, although heavily dependent on hydroelectricity, is beginning to explore the potential of wind and solar energy (Mosquera, 2023).

In Latin America, renewable energy sources have experienced significant growth, contributing 42.5% to the increase in generation capacity between 2010 and 2021. The region stands out for its abundant solar resource, especially evident in countries like Chile, Brazil, and Mexico, which are making considerable investments in solar energy. Additionally, wind energy has attracted investments in nations like Brazil, Chile, and Mexico. Hydroelectricity emerges as a key source of renewable energy in many Latin American countries, with Brazil being the largest producer with over 60% of its electricity coming from this source. Colombia and Peru also rely heavily on hydroelectricity. On the other hand, geothermal energy offers opportunities in countries like Chile and Mexico, with the latter possessing one of the world’s largest geothermal deposits. Finally, the use of biomass for energy generation is increasing, with nations like Brazil and Colombia investing in such projects to reduce their dependence on fossil fuels (Ongreso, 2023).

Large amounts of residual biomass generated in primary sectors such as agriculture and livestock are considered pollution in the linear economy view. However, these residues are valuable resources from the perspective of the circular green economy for new value chains. Economic agents involved in bioenergy in Latin America have recognized that there are prospects for cellulose ethanol production and in biomass conversion processes into energy vectors. However, bioenergy projects are diverse, and among others include established biomass producers in each country and consumers of biofuels and energy from biomass (IRENA, 2024).

The use of solar and wind energy in Latin America is conceived with the purpose of complementing hydroelectric and thermoelectric energy sources, in order to meet the growing energy demand of the countries (Hunt et al., 2022). Additionally, South American countries have the alternative of using residual biomass, since it does not generate significant emissions that affect the environment and can be obtained from various types of waste, as demonstrated in previous sections of this review. An example of this is Uruguay, where 18% of the energy used to generate electricity comes from biomass (Fornillo, 2021). Likewise, from RLB, as mentioned previously, it is possible to obtain energy, platform molecules, biofuels, and other materials for industrial use (Gómez Aguilar et al., 2020). Similarly, from other types of waste, it is feasible to obtain bioplastics, biomaterials, and recover proteins and enzymes for industrial use. Therefore, sustainable valorization of biomass represents both potential and a challenge for the development of the green economy.

The current situation and prospects of bioenergy in Latin America have been reviewed, highlighting important points to promote the effective implementation of sustainable systems for bioenergy production and use in the region, taking into account its own resources and needs. Among these points, the importance of developing relevant legal and regulatory frameworks stands out, as although it is true that the production of biofuels and bioelectricity is driven by the private sector, their regulation must be carried out by the governments of each country; the importance of implementing programs that allow the reduction of greenhouse gas emissions such as the one carried out with great success in Brazil (RenovaBio); the cooperation that must exist between Latin American countries in order to forge consensus on sustainability, availability, and governance of biomass, develop financing mechanisms, and promote guidance and convergence of policies (IRENA, 2024).

Finally, it is worth mentioning that there is interest from Latin American governments through policies to implement research projects to promote technological studies related to renewable energies and thus take advantage of all the interest that has been generated around these types of energies (Rueda-Bayona et al., 2019). The above, together with education and training at different levels of academic formation, are key to disseminate to the general public about these types of renewable energies and thus make an energy transition in the near future (Seminario-Córdova, 2023).

### Prospects for the future of RSB in South America

Based on the information reviewed and discussed in the different sections, it is evident that the LRB represents a surprising alternative for obtaining basic components and energy that can facilitate access to petroleum substitute products, particularly due to its great variety in South America. Although the region has numerous sources of biomass and favorable climatic conditions, it also faces certain limitations. By addressing these challenges and optimizing overall processes, the availability of more sustainable chemicals in the region can be improved. The biomass potential in South America countries is enormous and covers a wide range of resources, such as organic matter from municipal waste, forestry waste (e.g. bark, tree debris, wood chips) and agricultural waste (for example, corn stubble, vegetable stalks, wheat straw). In addition, favorable climatic conditions allow the cultivation of various biomass crops, including cereals in temperate zones and sugar cane or coffee in tropical zones.

The global demand for compounds derived from renewable biological sources is significant and encompasses the production of basic materials such as bioplastics, biopesticides, and biofertilizers, as well as solvents, biofuels (bioethanol, biogas, and biodiesel), and high-value products (cosmetics, pharmaceuticals, and biomaterials). Despite the potential, there are current limitations that must be addressed, including insufficient infrastructure, the need for technological advances, and regulatory frameworks. However, the evolution of this industry presents numerous opportunities, including attracting investment, increasing employment opportunities, and developing new infrastructure. In South America, academic research efforts abound, but initiatives by large companies also stand out, including Novozymes, Arauco, Braskem, Ecopetrol, and YPF, dedicated to the valorization of residual biomass.

Finally, mention that the use of biomass in the production of chemicals and biofuels has impressive potential to contribute to social, environmental and economic development in the South American region. Regional cooperation, the development of technologies, and the attraction of capital are undoubtedly the keys to success. A sustainable industry for South America is possible.

## Conclusions

Research on RLB in South America primarily focuses on characterization, conversion techniques, and biorefinery design. Brazil, Colombia, and Ecuador have reported the highest number of RLB sources, particularly sugarcane residues due to their abundance and high cellulose content. Various processes are employed for biofuel production, including dark fermentation and pyrolysis. Anaerobic digestion is common in Colombia and Argentina, while thermochemical processes such as pyrolysis and direct combustion have been extensively utilized in Brazil, Bolivia, Paraguay, Uruguay, and Ecuador. Solid fuels like pellets and briquettes have garnered significant attention for biomass valorization, with Venezuela focusing on briquette production and Chile on pellet production. Moreover, Peru and Paraguay have utilized oil residues for transesterification processes to produce biodiesel. Ethanol production follows a fermentative route, with alkaline pre-treatment and enzymatic hydrolysis being common. The production of HMF, FF, and LA has been documented in various countries, but the majority of investigations have been conducted in Brazil. Materials derived from RLB, such as activated carbon and nanomaterials, are gaining attention for environmental applications. Challenges exist in countries with limited RLB research, suggesting the need for policy support and international cooperation to leverage RLB’s potential as a sustainable energy source.

## Data Availability

Not applicable.
